# Evaluation of the Anti-Aging Properties of Ethanolic Extracts from Selected Plant Species and Propolis by Enzyme Inhibition Assays and 2D/3D Cell Culture Methods

**DOI:** 10.3390/ph18030439

**Published:** 2025-03-20

**Authors:** F. Sezer Senol Deniz, Ilkay Erdogan Orhan, Przemyslaw Andrzej Filipek, Abdulselam Ertas, Ronald Gstir, Thomas Jakschitz, Günther Karl Bonn

**Affiliations:** 1 Department of Pharmacognosy, Faculty of Pharmacy, Gazi University, 06330 Ankara, Türkiye; 2 Department of Pharmacognosy, Faculty of Pharmacy, Lokman Hekim University, 06510 Ankara, Türkiye; ilkay.erdoganorhan@lokmanhekim.edu.tr; 3 Principal Member of Turkish Academy of Sciences (TÜBA), Vedat Dalokay Street, No. 112, 06670 Ankara, Türkiye; 4 ADSI-Austrian Drug Screening Institute GmbH, Innrain 66a, 6020 Innsbruck, Austria; przemyslaw.filipek@adsi.ac.at (P.A.F.); ronald.gstir@i-med.ac.at (R.G.); thomas.jakschitz@adsi.ac.at (T.J.); guenther.bonn@uibk.ac.at (G.K.B.); 5 Department of Analytical Chemistry, Faculty of Pharmacy, Dicle University, 21280 Diyarbakır, Türkiye; abdulselamertas@hotmail.com

**Keywords:** cell culture, *Cotinus coggygria*, enzyme inhibition, phytocosmetic, propolis, *Vitis vinifera*

## Abstract

**Background**: Skin aging is a complex biological process affected by internal and external factors that disrupt the skin structure, especially in sun-exposed areas. Elastin and collagen in the dermis layer, responsible for the skin’s resistance and elasticity, have been the main subject of research. Since tyrosinase (TYR) is an enzyme found in different organisms and plays an essential role in melanogenesis, inhibitors of this enzyme have been the target mechanism for skin-bleaching product research. **Methods**: We selected the plant species *Cotinus coggygria* Scop., *Garcinia mangostana* L., *Pistacia vera* L., *Vitis vinifera* L., and propolis, which exhibited activity against a minimum of three target enzymes—elastase, collagenase, and TYR—in our previous screening study to find the suitable raw material for a cosmetic product. In the current research, the extracts from these samples were tested through a cell-free enzyme assay using validated elastase, collagenase, and TYR inhibition kits. We also performed the safety and efficacy tests of the selected extracts with 2D/3D cell culture methods. **Results**: Our data revealed the propolis extract among the tested ones displayed remarkable anti-inflammatory activity in the 2D (NF-κB induction: 10.81%) and 3D assays. *Cotinus coggygria* leaf and *Garcinia mangostana* shell extracts exhibited anti-inflammatory activity in the 2D luciferase reporter assay via TNFα addition. *C. coggygria* leaf, *V. vinifera* (grape) seed, and propolis extracts were selected for testing in 3D cell culture methods based on the 2D cytotoxicity results with cell viability values of 54.75%, 93.19%, and 98.64% at 34.25 µg/mL, respectively. The general phytochemical profiles of these three extracts were examined in terms of 53 phenolic compounds with LC-MS/MS, revealing that quinic acid, epicatechin, and acacetin were the dominant phenolics among the tested ones. **Conclusions**: It is the first study conducted to evaluate the use of the extracts indicated above in cosmetics by employing procedures involving 3D cell culture.

## 1. Introduction

Skin is one of the human body’s largest and most versatile organs, working as an immunogenic organ that acts as the first protective physical barrier and biological sensor against external allergens. It has additional functions such as regulating body temperature, controlling evaporation, and sensing and storing oil and water [1]. The skin ages due to two biological processes: intrinsic aging, where changes occur over a lifetime, and extrinsic aging, when changes are attributable to environmental and lifestyle factors. The primary external factors are infrared (IR) and ultraviolet (UV) exposure, causing photoaging, and smoking [2]. The skin produces free radicals and reactive oxygen species with repeated sun exposure, which causes oxidative stress and inflammatory responses in connective tissue’s dermal and epidermal layers [3]. Previous studies have also shown that in inflamed skin, such as photoaged skin, and with advancing age, matrix metalloproteinases (MMPs) are triggered, and dermal proteins such as collagen and elastin are destroyed with elastase and collagenase, resulting in skin damage and wrinkles [4,5,6]. Tyrosinase (TYR) is a copper-containing enzyme with monophenolase and diphenolase activity found in the membrane of melanosomes [7]. The first goal of developing reliable skin-lightening products is to search for TYR inhibitors, which take part in melanin biosynthesis [8,9]. Inhibition of elastase, collagenase, and TYR is frequently employed in the research of plant extracts and substances with dermo-cosmetic properties [10].

Due to their traditional medicinal use, plants represent a rich resource for developing potentially new drug candidates, many of which have yet to be discovered. In the same way, traditional plant practices can be used to formulate cosmetic products for skin care. Active herbal extracts and pure substances obtained from natural sources in cosmetic products have gained importance in recent years. So-called Natural cosmetics are trendy for consumers worldwide; the global market size for natural and organic cosmetics was USD 29.92 billion in 2021 [11]. Since cosmetic products come into contact with the skin almost daily, the reliability of raw materials is essential. However, most lack scientific evidence to verify their claimed cosmetic effects. Studies on plant extracts and natural compounds, which affect the activities of enzymes such as elastase, collagenase, and TYR, have been conducted in anti-aging natural cosmetic product research [12,13,14]. Inflammatory processes are well-known to accelerate skin aging, so discovering new compounds/extracts with anti-inflammatory properties is another target mechanism [15]. The use of animals in cosmetic research is not preferred due to increasing concerns about animal rights, as well as relevant ethical and scientific considerations. Their concerns encompass the pathogenic risk associated with certain derivatives and challenges related to preserving biodiversity and endangered species; they are substituted by phytochemicals, through chemical synthesis, or via biotechnological methods [16]. Due to ethical dilemmas, the European Union banned animal testing in cosmetic products and raw materials in March 2013 (https://ec.europa.eu/commission/presscorner/detail/en/ip_13_210, accessed on 14 March 2025). For this reason, studies on alternative methods have been conducted, and in recent years, three-dimensional (3D) skin models consisting of fibroblast and keratinocyte cell cultures have been successfully applied in cosmetic research [17]. Preliminary toxicological evaluation of new active compounds and herbal extracts can also be accomplished in skin cell lines such as keratinocytes and fibroblasts [18,19]. In addition to the safety tests, the anti-inflammatory effects of the samples can be easily detected in reconstructed human epidermis (RHE) models [20].

Our recent studies screened the inhibitory effect of approximately 100 extracts against elastase, collagenase, and TYR and their antioxidant activities using seven in vitro assays acting through different mechanisms [21]. We selected the plant species from this research, including *Cotinus coggygria* Scop., *Garcinia mangostana* L. (mangosteen), *Pistacia vera* L. (pistachio), and *Vitis vinifera* L. (grape), as well as propolis as an animal-based product, found to be effective against at least two target enzymes, for future investigations. The ethanolic extracts of the plant species mentioned were tested with a cell-free enzyme assay using validated assay kits to determine their elastase, collagenase, and TYR inhibitory capacity in the current study. First, the cytotoxicity, anti-inflammatory, and antioxidant activity of the extracts were tested in the HaCaT (human keratinocyte) cell line. The promising ones, such as *C. coggygria* leaf, grape seed, and propolis extracts, were advanced to further assays, including anti-inflammatory activity, skin tanning/whitening, and skin irritation/corrosion assays. Their activity was also performed using the RHE model.

*C. coggygria*, or the smoke tree, is a deciduous shrub esteemed for both its aesthetic appeal and its therapeutic benefits. This plant, indigenous to southern Europe and central China, has a longstanding history of application in traditional medicine owing to its varied bioactive components, such as flavonoids, tannins, and essential oils [22]. Recent investigations have revealed its potential therapeutic advantages, emphasizing its antibacterial, antioxidant, and anti-inflammatory properties. The flavonoid constituents in *C. coggygria* significantly influence these biological activities by regulating oxidative stress and suppressing proliferation [23]. Moreover, preparations from the leaves and bark have been utilized to address different diseases, including dermatological conditions, pharyngitis, and diarrhea [22,23]. *Vitis vinifera* L. (grape) seeds are a byproduct of the wine and fruit juice-producing industries. The predominant portion of the overall grape harvest is employed in vinification. The annual output capacity of grape seeds in Türkiye is approximately 30,000 tons [24]. Türkiye is also among the nations that produce and trade pistachios, and the outer shell of the pistachio, as a waste product, causes environmental issues. According to the average yield of the last five years, it is noted in the literature that approximately 20,000 tons of outer shells are produced annually in Türkiye [25]. The evaluation of these wastes generated in Türkiye is significant regarding the added value, which led us to include the extract from the grape seeds obtained from a winery in the Denizli region and pistachio shells from Gaziantep. Propolis is a resinous mixture that *Apis mellifera* (honeybee) collects from trees such as pine, oak, birch, eucalyptus, poplar, chestnut, etc., and the buds and leaves of some herbaceous plants to protect the hive and maintain its condition [26,27]. The color of the propolis may range from yellow-green to dark brown, depending on the plant varieties accessible to bees and the location of the collection [28]. This study aims to identify the optimal extract for formulating a successful anti-aging cosmetic by performing efficacy and safety tests in the 2D/3D cell culture of the extracts that demonstrated effectiveness in in vitro enzyme inhibition assays.

## 2. Results

### 2.1. Cell-Free Enzyme Assays

#### 2.1.1. TYR Inhibitory Activity

**Figure 1:**
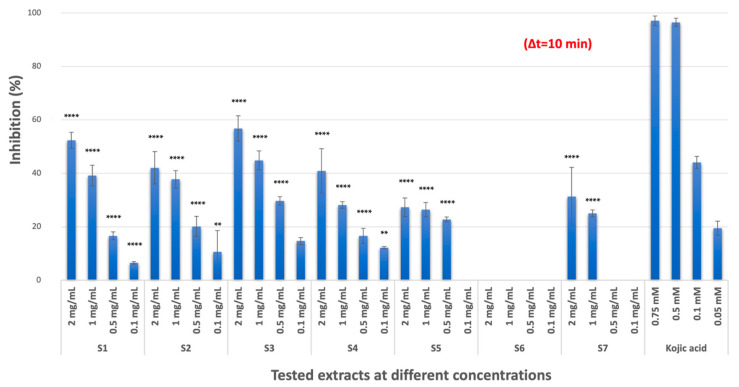
Relative TYR inhibitory activity of extracts at different concentrations (** *p* < 0.01, **** *p* < 0.0001).

*C. coggygria* extracts (S1, S2, and S3) and mangosteen extract (S4) had moderate TYR inhibition with inhibition values of 52.37%, 42.07%, 56.81%, and 40.89%, respectively (*p* < 0.0001, compared with positive control kojic acid) at the highest concentration (2 mg/mL stock concentration), while pistachio extract had no inhibition at the tested concentrations. Propolis (S5, 27.35%) and grape seed extract (S7, 31.32%) showed low inhibitory activity (*p* < 0.0001, compared with positive control kojic acid) against TYR (Figure 1). The results represented three independent experiments with a minimum of two technical replicates per experiment and are expressed as mean ± standard deviation (S.D.) (Table 1).

#### 2.1.2. Collagenase Inhibitory Activity

**Figure 2:**
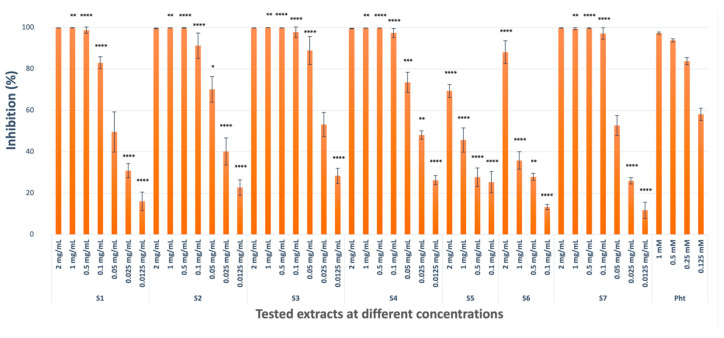
Collagenase inhibitory activities of the extracts at different concentrations (Pht: 1,10 phenanthroline, * *p* < 0.1, ** *p* < 0.01, *** *p* < 0.001, **** *p* < 0.0001).

*C. coggygria* extracts (S1, S2, and S3; 99.87%, 99.62%, and 99.88%), mangosteen extract (S4, 99.57%), and grape seed extract (S7, 99.78%) showed potent collagenase inhibitory potential (not significant compared with positive control 1,10 phenanthroline), where, therefore, 7 dilutions were prepared to calculate their IC_50_ values. Pistachio extract (S6, 88.09%) had significant inhibitory activity only at the highest concentration, while propolis extract (S5, 69.37%) had moderate inhibitory potential (*p* < 0.0001 compared with positive control 1,10 phenanthroline) against collagenase (Figure 2). The results represented three independent experiments with a minimum of two technical replicates per experiment and are expressed as mean ± S.D. (Table 1).

#### 2.1.3. Elastase Inhibitory Activity

**Figure 3:**
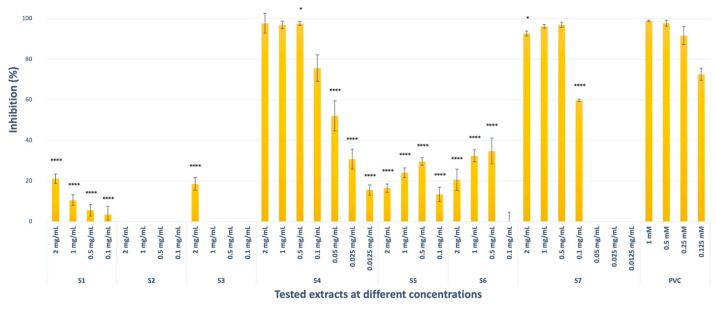
Elastase inhibitory activities of the extracts at different concentrations (PVC: *N*-Methoxysuccinyl-Ala-Ala-Pro-Val-chloromethyl ketone, * *p* < 0.1, **** *p* < 0.0001).

Mangosteen (S4, 97.73%, not significant compared with positive control *N*-Methoxysuccinyl-Ala-Ala-Pro-Val-chloromethyl ketone) and grape seed (S7, 92.70%, *p* < 0.1 compared with positive control *N*-Methoxysuccinyl-Ala-Ala-Pro-Val-chloromethyl ketone) extracts showed potent inhibitory activity against elastase at the highest concentrations. *C. coggygria* commercial extract (S1, 21.09%) and leaf extract (S3, 18.54%) had moderate inhibitory potential (*p* < 0.0001 compared with positive control *N*-Methoxysuccinyl-Ala-Ala-Pro-Val-chloromethyl ketone) at 2 mg/mL stock concentration, while pedicel extract (S2) was inactive at the tested concentrations. Propolis (S5, 16.48%) and pistachio (S6, 20.62%) extracts also displayed moderate elastase inhibitory activity (*p* < 0.0001) as compared to that of *N*-Methoxysuccinyl-Ala-Ala-Pro-Val-chloromethyl ketone, used as a positive control. The elastase inhibition by S5 and S6 was not in a concentration-dependent manner for any of the replicates (Figure 3). The results represented three independent experiments with a minimum of two technical replicates per experiment and are expressed as mean ± S.D. (Table 1).

### 2.2. Cell-Based Assays

#### 2.2.1. Cytotoxicity Assay

**Figure 4:**
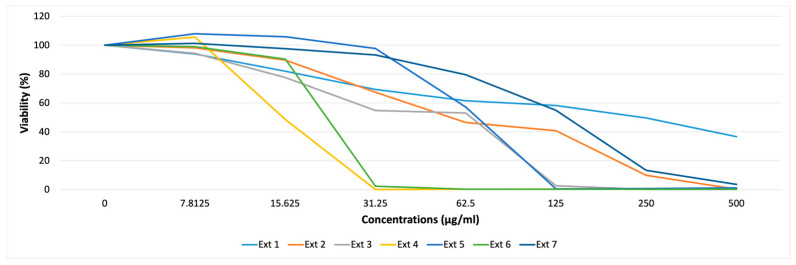
Percentages of cell viability using a resazurin assay where HaCaT cells were exposed to increasing concentrations of extracts for 24 h.

The extracts were analyzed for their possible cytotoxicity against HaCaT cell lines using a resazurin assay. Our results indicated that mangosteen (S4, 0.12% at 31.25 µg/mL) and pistachio (S6, 2.33% at 31.25 µg/mL) extracts exhibited a significant reduction in the number of viable cells at a concentration higher than 15.625 µg/mL (Figure 4). The results are representative of at least three independent experiments with three technical replicates per experiment.

#### 2.2.2. Anti-Inflammatory and Antioxidant Activity

Mangosteen (S4, 5.91% at 15 µg/mL) and propolis (S5, 10.81% at 50 µg/mL) extracts possessed anti-inflammatory activity in a concentration-dependent manner, while *C. coggygria* leaf extract (S3, 25.10%) had anti-inflammatory activity at the highest concentration (25 µg/mL) with tumor necrosis factor-*alpha* (TNFα, 1 ng/mL) addition (Figures S1 and S2). *C. coggygria* pedicel extract (S2) decreased the nuclear factor kappa B (NF-κB) activity both pre- and post-treatment with UV-B (81.55% and 49.46% at 25 µg/mL, respectively), while mangosteen (S4, 57.52% at 15 µg/mL), propolis (S5, 39 97% at 50 µg/mL), and pistachio shell extract (S6, 54.94% at 15 µg/mL) decreased NF-κB induction only post-treatment. Grape seed extract (S7) increased the NF-κB induction both pre- and post-treatment (230.25% and 152.65% at 50 µg/mL, respectively) in a dose-dependent manner (Figures S3–S6). Mangosteen (S4, 70.44% at 15 µg/mL), propolis (S5, 19.37% at 50 µg/mL), and pistachio (S6, 45.40% 15 µg/mL) extracts had antioxidant activity against H_2_O_2_ stimulation (Figures S7 and S8). Based on the cytotoxicity, anti-inflammatory, and cell-free enzyme results, *C. coggygria* leaf (S3), propolis (S5), and grape seed extracts (S7) were selected for further studies using 3D skin model methods.

#### 2.2.3. Anti-Inflammatory Activity on Reconstructed Human Epidermis (RHE)

Considering the decreased amount of the chemokines MPC-1 and RANTES, propolis (S5, 384.25/141 and 117.63/729, respectively, after 48 h at 0.5 mg/mL) and grape seed extracts (S7, 1626/451 and 13491.5/1492, after 48 h at 0.5 mg/mL) displayed anti-inflammatory activity in a concentration-dependent manner on RHE (Figure S9). These chemokines related to skin regeneration were selected in this assay. We tested three extracts at three different concentrations in two independent experiments. Based on the results, we decided to perform two more replicates for propolis (S5) and grape seed extracts (S7) (Figure S10).

#### 2.2.4. Skin Tanning/Whitening Assay

No significant or reproducible whitening/tanning effects were observed for the *C. coggygria* leaf (S3), propolis (S5), or grape seed extracts (S7) on the epiCS-M at the tested concentrations (Figures S11–S13).

#### 2.2.5. Skin Irritation/Corrosion Test

**Figure 5:**
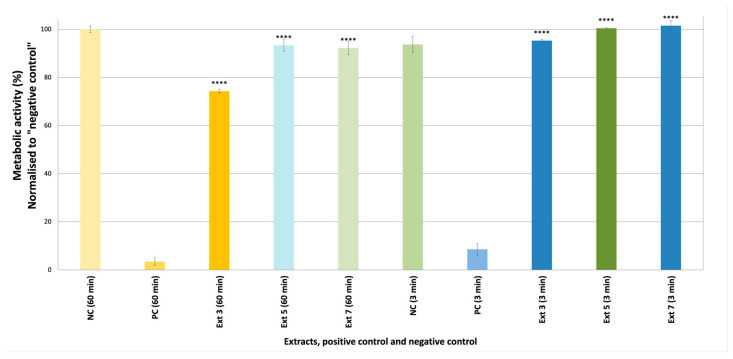
The metabolic activity values (%) of the epiCS treated with S3, S5, and S7 after 3 and 60 min [Negative control (NC): H_2_O, Positive control (PC): 8M KOH, *n* = 2], **** *p* < 0.0001.

**Figure 6:**
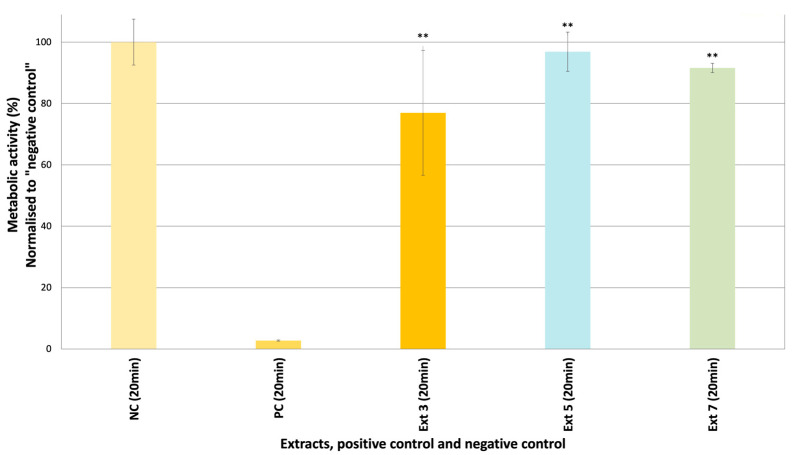
The metabolic activity values (%) of the epiCS treated with S3, S5, and S7 after 20 min [Negative control (NC): DPBS, Positive control (PC): 5% SDS, *n* = 2], ** *p* < 0.01.

Skin irritation/corrosion tests on epiCS are the first steps in the safety assessment of active extracts. The skin irritation and corrosion potentials of *C. coggygria* leaf (S3), propolis (S5), and grape seed extracts (S7) were tested in epiCS. Based on the 3-(4,5-dimethylthiazol-2-yl)-5-(3-carboxymethoxyphenyl)-2-(4-sulfophenyl)-2H-tetrazolium (MTT) results obtained herein, none of the extracts were either corrosive or irritative (Figure 5 and Figure 6). The cell viability values were lower in epiCS treated with S3 (74.28%) than the epiCS treated with other extracts (S5: 93.33%, S7: 93.27%).

### 2.3. LC-MS/MS Analysis

**Figure 7:**
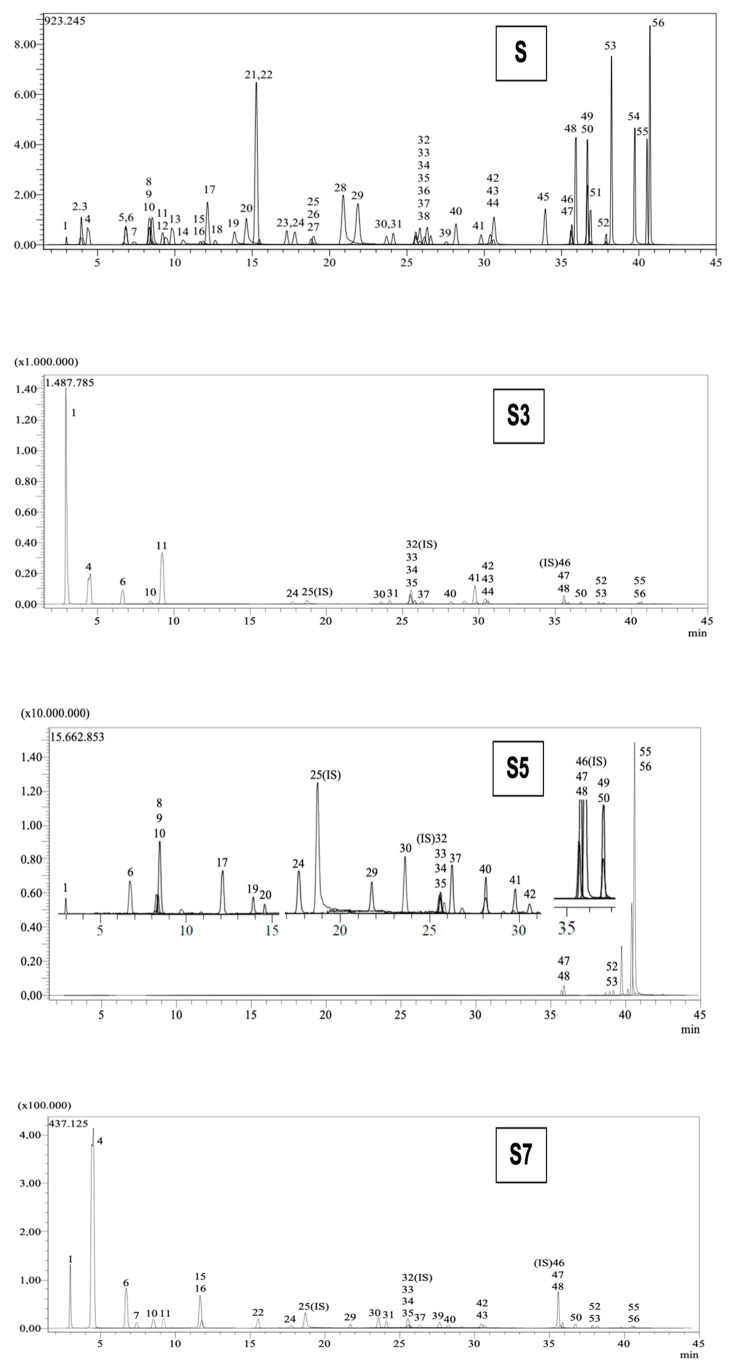
LC chromatogram of the standards (S) and extracts (S3, S5, and S7). (1: Quinic acid. 2: Fumaric acid. 3: Aconitic acid. 4: Gallic acid. 5: Epigallocatechin. 6: Protocatechuic acid. 7: Catechin. 8: Gentisic acid. 9: Chlorogenic acid. 10: Protocatechuic aldehyde. 11: Tannic acid. 12: Epigallocatechingallate. 13: 1,5-dicaffeoylquinic acid. 14: 4-OH Benzoic acid. 15: Epicatechin. 16: Vanilic acid. 17: Caffeic acid. 18: Syringic acid. 19: Vanillin. 20: Syringic aldehyde. 21: Daidzin. 22: Epicatechingallate. 23: Piceid. 24: *p*-Coumaric acid. 26: Ferulic acid. 27: Sinapic acid. 28: Coumarin. 29: Salicylic acid. 30: Cynaroside. 31: Miquelianin. 33: Rutin. 34: isoquercitrin. 35: Hesperidin. 36: *o*-Coumaric acid. 37: Genistin. 38: Rosmarinic acid. 39: Ellagic acid. 40: Cosmosiin. 41: Quercitrin. 42: Astragalin. 43: Nicotiflorin. 44: Fisetin. 45: Daidzein. 47: Quercetin. 48: Naringenin. 49: Hesperetin. 50: Luteolin. 51: Genistein. 52: Kaempferol. 53: Apigenin. 54: Amentoflavone. 55: Chrysin. 56: Acacetin.).

Table 2 tabulates the identified phenolic compounds and their amounts (mg/g extract) in *C. coggygria* leaf (S3), propolis (S5), and grape seed (S7) extracts. Among the 53 standard phenolic compounds available in our MS library, quinic acid for *C. coggygria* leaf (126.686 mg/g extract), acacetin for propolis (62.332 mg/g extract), and epicatechin for grape seed extracts (45.26 mg/g extract) were detected as the most abundant phenolic compounds. The LC chromatograms of S3, S5, and S7 are provided in Figure 7.

## 3. Discussion

Plant extracts and compounds originating from natural sources are increasingly attracting interest for their potential application in the cosmetic industry. Nevertheless, additional investigation is imperative to comprehensively comprehend the biological activity, safety, and exact method of action of these raw materials, as well as to identify and describe the active compounds accountable for these activities. Following the implementation of stringent European Union laws that prohibited animal trials entirely, experts in the cosmetic industry have turned their attention to utilizing cultivated primary cells in either two-dimensional or three-dimensional formats. Based on our substantial in vitro screening results, we selected some extracts for further studies to determine their effects on 2D/3D cell cultures [21].

Based on its traditional usage against skin disorders in Türkiye, we selected *C. coggygria* for activity-guided fractionation, and we previously reported isolation of astragalin (kaempferol-3-*O*-glucoside), hyperoside (quercetin-3-*O*-galactoside), isoquercitrin (quercetin-3-*O*-glucoside), and methyl gallate from the active fractions against cosmetic-related enzymes [29]. In this study, we tested three *C. coggygria* extracts (leaf, pedicel, and commercial extract), and the leaf extract (S3) was selected for further studies based on the cytotoxicity results. The leaf extract of the plant is registered as an astringent, antioxidant, and humectant in the cosmetic ingredient (CosIng) database (https://ec.europa.eu/growth/tools-databases/cosing/details/96188, accessed on 14 March 2025); however, its essential oil from the branches and leaves is more commonly used in cosmetic products. We have conducted the first investigation into the effective and safe application of the plant’s leaf extract as a cosmetic raw material. According to our data acquired through the LC-MS/MS analyses, quinic acid is the most abundant phenolic compound in the leaf extract, quantified at 129.686 mg/g extract. Studies have shown that the compound exhibits antioxidant, antimicrobial, anticancer, antiviral, antidiabetic, antinociceptive, and analgesic effects [30]. In recent studies, quinic acid displayed anti-inflammatory activity acting through the inhibition of the expression of proinflammatory mediators and the oxidative stress marker in a dose-dependent manner in the lipopolysaccharide (LPS)-injected mice hippocampus, and suppression of the TLR4-NF-κB and NF-κB-iNOS-NO signaling pathways in Wistar rats [31,32]. In a clinical study, oral administration of quinic acid (1000 mg/day for 60 days) significantly improved DNA repair and lifestyle-induced clinical responses, including skin quality [33]. Nevertheless, the limited bioavailability of quinic acid is of great importance when developing innovative formulations, and this should be noted [30].

The chemical composition of propolis varies according to the source of the plant, region, and season, but generally consists of resin (50%), wax (30%), essential oil (10%), pollen (5%), and other organic compounds (5%) in average. Propolis, which was reportedly used even in ancient Egypt, has marked pharmacological effects such as anti-inflammatory, wound healing, antioxidant, neuroprotective, hypotensive, immune system stimulant, bacteriostatic, and bactericidal effects [34,35]. Secondary metabolites responsible for biological activities are phenolic and aromatic compounds, including flavonoids [36,37]. Different bee products, such as honey, royal jelly, beeswax, bee venom, bee pollen, and propolis, are frequently used in cosmetic product formulations as active ingredients [38]. In the commercial ethanol extract of propolis sample (S5), acacetin (5,7-dihydroxy-4-methoxyflavone), an *O*-methylated flavone, was the predominant phenolic compound (62.332 mg/g extract). Recent scientific research has demonstrated the efficacy of acacetin in addressing several conditions, including inflammation, arthritis, obesity, diabetes, and viral infections through in vitro systems [39]. Studies indicate that acacetin can proficiently regulate the inflammatory response by obstructing critical signaling pathways, including NF-κB and MAPK, which are implicated in releasing pro-inflammatory cytokines and enzymes such as cyclooxygenase-2 (COX-2) and inducible nitric oxide synthase (iNOS). Acacetin diminishes the generation of inflammatory mediators, mitigating symptoms related to inflammatory illnesses. Moreover, acacetin’s antioxidant characteristics enhance its anti-inflammatory benefits by neutralizing free radicals that may intensify inflammation [40,41,42]. Due to its various actions, acacetin serves as an excellent multifunctional raw material for cosmetics, and various propolis samples from diverse origins can be considered for their phytocosmetic potential [37].

The other active extract was *Vitis vinifera* seed extract, a well-known cosmetic ingredient. *Vitis vinifera*, or the grapevine, is renowned for its seeds, which are abundant in phenolic compounds, especially proanthocyanidins and flavonoids, making them valuable in cosmetic formulations. These active compounds are recognized for their powerful antioxidant activities, safeguarding the skin against oxidative stress and damage induced by environmental factors such as UV radiation and pollution. Moreover, grape seed extract exhibits anti-inflammatory properties, facilitating skin soothing and enhancing complexion health [43]. Based on the LC-MS/MS analyses in this study, epicatechin (45.26 mg/g extract), gallic acid (16.901 mg/g extract), and quinic acid (11.382 mg/g extract) were found to be the major compounds of the grape seed extract. Recent findings demonstrate that polyphenols can diminish inflammatory reactions by directly interacting with proteins that initiate the response. These phenolic compounds may be responsible for the antioxidant and anti-inflammatory activities identified in the extract [44,45].

Although the utilization of plants and natural compounds for cosmetic applications has a history spanning 5000 years [46], today, contemporary scientific data supporting many of these substances remain inadequate, rendering their benefits speculative. Natural extracts have significant benefits for skin care; nevertheless, additional research and clinical evidence are necessary, as the efficacy of many of these extracts remains contentious [47,48]. This study aimed to evaluate the efficacy of extracts, some utilized in cosmetic formulations, through 3D cell culture techniques and to establish a scientific-based cosmetic product in the future.

## 4. Materials and Methods

### 4.1. Plant Materials and Extraction Procedure

The air-dried plant parts were grounded in a mechanic grinder into a fine powder and weighed precisely (15 gr) on a digital balance (Shimadzu, Kyoto, Japan). Each plant part was extracted with 250 mL ethanol (EtOH, 80%) for 5 days and shaken by hand occasionally at room temperature. The organic phases were filtered and evaporated under a vacuum using a rotary evaporator (Buchi, Flawil, Switzerland) to obtain the crude extracts. The extracts were stored at 4 °C until the experiments were performed. The collection (procurement for commercial ones) sites and dates of the plant species screened in this study and the extract yields (%, *w*/*w*) are tabulated in Table 3. The voucher specimens of the collected plant samples were stored at the Herbarium of the Faculty of Pharmacy, Gazi University (Ankara, Türkiye) [19].

### 4.2. Cell-Free Enzyme Assays

#### 4.2.1. TYR Inhibitory Activity

The TYR inhibitory potential of the extracts was tested with anti-TYR assay kits (Abcam, Cambridge, UK, ab204715, #GR3317459-1) at different concentrations. The method is based on the catalyzing function of TYR (EC 1.14.18.1) on the oxidation of tyrosine, producing a chromophore that can be detected at 510 nm. *Alpha*-kojic acid, a reversible TYR inhibitor, was used as a positive control. The resulting increase in fluorescence was monitored with a fluorescence microplate reader (Tecan Spark, Männedorf, Switzerland). GraphPad Prism 6 program was used to calculate the IC_50_ values.

#### 4.2.2. Collagenase Inhibitory Activity

The collagenase inhibitory potential of the extracts was tested with an anti-collagenase assay kit (Thermo Fischer Scientific, Waltham, MA, USA, E12055, #2098879) at different concentrations. The kit contains DQ™—gelatin substrate (from pig), which has been heavily labeled with fluorescein, so the conjugate’s fluorescence is quenched. The non-fluorescent substrate can be digested by gelatinase and collagenase from *Clostridium histolyticum*. The resulting increase in fluorescence was monitored with a fluorescence microplate reader (Tecan Spark, Männedorf, Switzerland). Digestion products from the DQ gelatin substrate have absorption maxima at ~485 nm and fluorescence emission maxima at ~530 nm. The kit includes a selective inhibitor of collagenase, 1,10-phenanthroline monohydrate, which was used as a positive control. The GraphPad Prism 6 program was used to calculate the IC_50_ values.

#### 4.2.3. Elastase Inhibitory Activity

The elastase inhibitory potential of the extracts was tested with an anti-elastase assay kit (Thermo Fischer Scientific, Waltham, MA, USA, E12056, #2146719) at different concentrations. The kit contains DQ™ elastin—soluble bovine neck ligament elastin labeled with BODIPY^®^ FL dye, so the conjugate’s fluorescence is quenched. The non-fluorescent substrate can be digested by elastase from the pig pancreas. The resulting increase in fluorescence was monitored with a fluorescence microplate reader (Tecan Spark, Männedorf, Switzerland). Digestion products from the DQ elastin substrate have absorption maxima at ~505 nm and fluorescence emission maxima at ~515 nm. The kit includes a selective inhibitor of elastase, *N*-methoxysuccinyl-Ala-Ala-Pro-Val-chloromethyl ketone, which was used as a positive control. The GraphPad Prism 6 program was used to calculate the IC_50_ values.

### 4.3. Cell-Based Assays

#### 4.3.1. Cell Culture

Cytotoxic studies were determined in the HaCaT (human keratinocyte, AddexBio, Geneva, Switzerland, #T0020001) cell line. The cells were cultured in Dulbecco’s Modified Eagle Medium (DMEM) (Gibco, Grand Island, New York, NY, USA) enriched with 10% fetal bovine serum (FBS) and sodium pyruvate under a humidified 5% CO_2_ atmosphere at 37 °C. FBS (2%) was used instead of 10% for the treatment with the extracts in the anti-inflammatory, antioxidant activity, and cytotoxicity assays. HaCaT NF-κB Luc Bulk cell lines [49] were used for the luciferase-based NF-κB reporter assay (inflammation stimulus = UV-B irradiation, oxidative stress stimulus = H_2_O_2_). The HaCaT NF-κB Luc Bulk cells were processed identically as the parental cell line with two exceptions; 1 µg/mL puromycin (Thermo Fisher Scientific, Waltham, MA, USA, #A1113803) was added to the medium to apply a selection pressure on the cells, and the cell numbers for splitting were different.

#### 4.3.2. Cytotoxicity Assay

Cell viability was examined with a resazurin assay. Resazurin is a cell-permeable redox indicator that can monitor viable cell numbers with protocols similar to those utilizing tetrazolium compounds. Resazurin can be dissolved in physiological buffers (resulting in a deep-blue-colored solution) and added directly to cells in culture in a homogeneous format. Viable cells with active metabolisms can reduce resazurin into the resorufin product, which is pink and fluorescent. In all treatments, the extracts were dissolved initially in DMSO and made up to the required concentration with a complete cell culture medium (final maximum concentration of DMSO 0.5% *v*/*v*). The extract dilutions were incubated overnight. The resazurin sodium salt (Sigma-Aldrich, St. Louis, MO, USA) was dissolved in DPBS (pH 7.4) to 1.5 mg/mL, and a 1:100 dilution was prepared from this stock solution with the HaCaT medium. Approximately 100 µL of diluted resazurin solution was added to each well, and the plates were incubated for two hours at 37 °C. The fluorescence was recorded at 560 nm excitation/590 nm emission with a multimode microplate reader (Tecan Spark, Männedorf, Switzerland).

#### 4.3.3. Anti-Inflammatory and Antioxidant Activity

The luciferase reporter assay was utilized to determine the activation of NF-κB in HaCaT NF-κB Luc cells induced *via* TNFα (1 ng/mL) addition or UV-B irradiation to screen the anti-inflammatory properties of the extracts. Two parallel experiments were employed for the UV-B stimulus. The extracts were added to one plate, and after incubation, the cells were induced with UV-B at 0.15 J/cm^2^ (pre-treatment). In the parallel experiment, the cells were induced with UV-B as 0.15 J/cm^2^, and then the extracts were added to the microplate at indicated concentrations (post-treatment). H_2_O_2_ was used as an oxidative stress stimulus to determine the antioxidant capacity of the extracts. Cells were treated with non-cytotoxic concentrations of the extracts, and NF-κB reporter activity was quantified with a luciferase bioluminescence assay. Since withaferin A (WFA) directly obstructs NF-κB activation by inhibiting p65 dimerization; it was used as the NF-κB inhibitory control. The cell viability was monitored using the resazurin assay.

#### 4.3.4. Anti-Inflammatory Activity on Reconstructed Human Epidermis (RHE)

The anti-inflammatory capacity of the selected extracts was evaluated with TNFα-induced inflammation of the RHE. The “epiCS” (Phenion, Düsseldorf, Germany, #CS1001) inserts with epidermis tissues (origin human keratinocytes) were used to determine the effect of the extracts on skin inflammation. Therefore, the selected extracts were added to the medium and pre-incubated for 1 h. Then, inflammation was induced by TNFα. After 24 h, the TNFα, extracts, and controls were removed. Subnatants of the RHE models were harvested 24 and 48 h after treatment to analyze the cytokine expression using the custom human cytokine magnetic 10-Plex kit (Bio-Techne, Minneapolis, MN, USA, LXSAHM-10). Furthermore, a first impression of the condition/the viability of the human epidermis equivalents was received 24 h after treatment via the colorimetric CytoTox 96 (Promega, Madison, WI, USA, #G1780) assay. This assay measures the amount of cytosolic lactate dehydrogenase (LDH), which is passively released from cells into the medium upon damage to the cell membrane and, thus, is a measure for cell death. Approximately 48 h after treatment, the metabolic activity of the cells was analyzed using MTT.

#### 4.3.5. Skin Tanning/Whitening Assay

Human epidermis equivalents with melanocytes (epiCS-M, Phenion, Düsseldorf, Germany, #CS1101) were used to determine the effect of the extracts on skin tanning. The selected extracts were applied in the medium of the epiCS-M over 14 days at the maximum. During exposure of the epiCS-M to the extracts, cell damage was monitored using the colorimetric CytoTox 96 (Promega, Madison, WI, USA, #G1780) assay. At the endpoint of the experiment (after 14 days of the cultivation and treatment of epiCS-M), the skin tanning status of each epiCS-M was documented photographically and analyzed using ImageJ (version 1.52g) software. Finally, the metabolic activity of the epiCS-M was analyzed using MTT. A known stimulator of melanogenesis, 3-Isobutyl-1-methylxanthine (IBMX), was used as the skin tanning control, while 4-butylresorcinol, an inhibitor of melanogenesis, served as the skin whitening control. Since the final concentration of the DMSO should not be higher than 1%, the extracts were tested at 10, 20, and 30 µg/mL concentrations.

#### 4.3.6. Skin Irritation Test

The skin irritation test using epiCS provides a prediction about reversible skin damage. The epiCS (Phenion) model consists of normal, human-derived epidermal keratinocytes, which have been cultured to form a multilayered, highly differentiated model of the human epidermis. It consists of organized basal, spinous, and granular layers and a multilayered stratum corneum containing intercellular lamellar lipid layers arranged in patterns analogous to those found in vivo. On the day of receipt, the epiCS were conditioned by incubation in an epiCS culture medium to release transport-stress-related compounds and debris for at least 4 h or overnight. After pre-incubation, the tissues were topically exposed to the selected extracts, positive control (5% sodium dodecyl sulfate), and negative control (DPBS) for 20 min. Tissues were then thoroughly rinsed with 1xDPBS, blotted to remove the test substances/controls, and transferred to a fresh medium. After a 24 h incubation period, the medium was changed, and the tissues were incubated for another 18 h. Afterward, an MTT assay was performed by transferring the tissues to 24 well plates containing 300 µL MTT medium (1 mg/mL). After 3 h of MTT incubation, the blue formazan salt formed by cellular mitochondria was extracted with isopropanol (2 mL) per tissue, and the optical density of the extracted formazan was determined in a spectrophotometer at 570 nm. Relative cell viability is calculated for each tissue as % of the mean of the negative control tissues. The skin irritation potential of the test materials is predicted if the mean relative cell viability of the tissue is below or equal to 50%. According to the United Nations Globally Harmonized System (UN-GHS), for classification and labeling, the test protocol allows for predicting the skin irritation potential of test substances (https://unece.org/transport/dangerous-goods/ghs-rev10-2023, accessed on 14 March 2025). A reduction of tissue viability equal to or below 50% of the negative control classifies the substances as “category 2”. Tissue viability of above 50% is classified as “no category”.

#### 4.3.7. Skin Corrosion Test

The skin corrosion test using epiCS provides a prediction about unreversible skin damage. The epiCS tissues (Phenion, surface 0.6 cm^2^) were conditioned pre-incubation in an epiCS culture medium to release transport stress-related compounds and debris. After overnight pre-incubation, the tissues were transferred to a fresh epiCS culture medium and topically exposed with the selected extracts, positive control (8M KOH), and negative control (H_2_O) for 3 min and 1 h, respectively. After exposure, tissues were rinsed and blotted, and an MTT assay medium replaced the culture medium. After 3 h incubation, tissues were transferred to 2 mL isopropanol, and the blue formazan salt was extracted with isopropanol. The optical density of the formazan extract was determined spectrophotometrically at 540–570 nm, and cell viability was calculated for each tissue as % of the mean of the negative control tissues. The skin corrosivity potential of the test materials was classified according to the remaining cell viability obtained after 3 min or 1 h of exposure to the extracts. The test protocol allows for the prediction of the skin corrosivity potential of test substances according to the UN-GHS for classification and labeling. The prediction model works in two steps (Table 4).

### 4.4. Statistical Analyses

The enzyme inhibitory effects were statistically examined using one-way ANOVA, followed by Dunnett’s multiple comparison test, to compare the positive control with the test groups. The cell-based assay results were statistically analyzed using one-way ANOVA, followed by Dunnett’s multiple comparison test, to compare the negative control with the test groups (GraphPad Prism 6.01). Values of *p* ≤ 0.05 were regarded as statistically significant.

### 4.5. LC-MS/MS Analyses

A Shimadzu-Nexera model ultrahigh performance liquid chromatography (UHPLC), coupled with a tandem mass spectrometer, was used to evaluate 56 phytochemicals quantitatively. The reversed-phase UHPLC was equipped with an autosampler (SIL-30AC model), a column oven (CTO-10ASvp model), binary pumps (LC-30AD model), and a degasser (DGU-20A3R model). The chromatographic conditions were optimized to achieve optimum separation for 56 phytochemicals and overcome the suppression effects. Different columns such as the Agilent Poroshell 120 EC-C18 model (150 mm × 2.1 mm, 2.7 µm) and RP-C18 Inertsil ODS-4 (100 mm × 2.1 mm, 2 µm), different mobile phases (B) such as acetonitrile and methanol, different mobile phase additives such as ammonium formate, formic acid, ammonium acetate, and acetic acid, and different column temperatures such as 25 °C, 30 °C, 35 °C, and 40 °C were tried and applied until the optimum conditions were achieved. Consequently, the chromatographic separation was performed on a reversed-phase Agilent Poroshell 120 EC-C18 model (150 mm × 2.1 mm, 2.7 µm) analytical column. The column temperature was set to 40 °C. The elution gradient comprised eluent A (water + 5 mM ammonium formate + 0.1% formic acid) and eluent B (methanol + 5 mM ammonium formate + 0.1% formic acid). The following gradient elution profile was used: 20–100% B (0–25 min), 100% B (25–35 min), 20% B (35–45 min). Furthermore, the solvent flow rate and injection volume were settled as 0.5 mL/min and 5 µL, respectively.

The mass spectrometric (MS) detection was performed using a Shimadzu LCMS-8040 model tandem mass spectrometer with an electrospray ionization (ESI) source operating in negative and positive ionization modes. LC-ESI-MS/MS data were acquired and processed by LabSolutions (version 5.97) software (Shimadzu, Kyoto, Japan). The multiple reaction monitoring (MRM) mode was used to quantify the metabolites. The MRM method was optimized to selectively detect and quantify phytochemical compounds based on screening-specified precursor metabolite-to-fragment ion transitions. The collision energies (CEs) were optimized to generate optimal phytochemical fragmentation and maximal transmission of the desired product ions. The MS operating conditions were applied as drying gas (N_2_) flow, 15 L/min; nebulizing gas (N_2_) flow, 3 L/min; DL temperature, 250 °C; heat block temperature, 400 °C; and interface temperature, 350 °C [50].

## 5. Conclusions

From our cumulative results, it can be concluded that propolis, among the tested extracts, displayed remarkable anti-inflammatory activity in our 2D and 3D assays and can be proposed as an active ingredient in the formulation of a scientifically approved and natural-based dermocosmetic product. It is also possible to test propolis samples of different origins obtained from various plant sources. Our data revealed that *C. coggygria* leaf extract and mangosteen have anti-inflammatory activity in the 2D luciferase reporter assay via TNFα addition. Mangosteen and pistachio shell extracts exhibited significant anti-inflammatory activity in the 2D luciferase reporter assay with UV-B irradiation and antioxidant activity through H_2_O_2_ stimulation. On the other hand, we could not test these extracts on 3D models because of the high cytotoxicity and low cell viability results from the 2D *in vitro* tests and limitations due to the final concentration of DMSO. Even so, it can be suggested as an option to prepare the refined extracts to eliminate the possible adverse effects of the cytotoxic compounds in the extracts, such as mangosteen and pistachio shell, as they are efficient against inflammation. The active plant species in the cell-free enzyme assays were observed to overlap with their traditional usages, which primarily have been recorded against skin disorders and inflammation. This has been the first study to investigate the application of the extracts mentioned above in cosmetics using 3D cell culture techniques. Although some of the plant species in this study, such as grape seed and propolis, are commonly used in cosmetics, their effectiveness as a cosmetic active ingredient through some mechanisms was scientifically reported for the first time in the present work. Further studies are required to analyze different propolis samples from diverse origins to compare their activities and formulate an innovative anti-aging product using *C. coggygria* extract.

## Figures and Tables

**Table 1:** TYR, collagenase, and elastase inhibitory effects and the IC_50_ values of the extracts.

Codes	TYR Enzyme Inhibition (Inhibition % ± S.D. ^a^) 2 mg/mL ^b^	Collagenase Enzyme Inhibition (Inhibition % ± S.D. ^a^) 2 mg/mL ^b^	Elastase Enzyme Inhibition (Inhibition % ± S.D. ^a^) 2 mg/mL ^b^
S1	52.37 ± 2.99 **** (IC_50_ = 1.70 ± 0.02 mg/mL)	99.87 ± 0.03 (IC_50_ = 0.043 ± 0.003 mg/mL)	21.09 ±2.28 ****
S2	42.07 ± 6.07 ****	99.62 ± 0.22 (IC_50_ = 0.030 ± 0.004 mg/mL)	- ^c^
S3	56.81 ± 4.77 **** (IC_50_ = 1.52 ± 0.12 mg/mL)	99.88 ± 0.01 (IC_50_ = 0.022 ± 0.001 mg/mL)	18.54 ± 3.11 ****
S4	40.89 ± 8.30 ****	99.57 ± 0.12 (IC_50_ = 0.025 ± 0.001 mg/mL)	97.73 ± 4.85 (IC_50_ = 0.045 ± 0.005 mg/mL)
S5	27.35 ± 3.46 ****	69.37 ± 3.07 **** (IC_50_ = 1.08 ± 0.19 mg/mL)	16.48 ± 2.07 ****
S6	-	88.09 ± 5.51 **** (IC_50_ = 1.11 ± 0.05 mg/mL)	20.62 ± 5.26 ****
S7	31.32 ± 10.93 ****	99.78 ± 0.08 (IC_50_ = 0.041 ± 0.001 mg/mL)	92.70 ± 1.24 * (IC_50_ = 0.076 ± 0.006 mg/mL)
REF	97.08 ± 1.79 ^d^ (IC_50_ = 0.11 ± 0.01 mM)	97.32 ± 0.52 ^e^ (IC_50_ = 0.103 ± 0.009 mM)	98.87 ± 0.43 ^f^ (IC_50_ = 0.156 ± 0.03 mM)

^a^ Standard deviation (*n* = 6), ^b^ Stock concentration, ^c^ No activity, ^d^ *Alpha*-kojic acid (0.75 mM), ^e^ 1,10 phenanthroline 1 mM, ^f^ *N*-Methoxysuccinyl-Ala-Ala-Pro-Val-chloromethyl ketone (1 mM), * *p* < 0.05, **** *p* < 0.0001.

**Table 2:** Identification and quantification of phenolic compounds of *Cotinus coggygria* leaf, *Vitis vinifera* seed, and propolis extracts by LC-MS/MS.

No	Analytes	RT ^a^	M.I. (*m*/*z*) ^b^	F.I. (*m*/*z*) ^c^	Ion. Mode	Equation	*r^2^* ^d^	*Cotinus coggygria* Leaf (mg Analyte/g Extract)	*Vitis vinifera* Seed (mg Analyte/g Extract)	Propolis (mg Analyte/g Extract)
1	Quinic acid	3.0	190.8	93.0	Neg	*y* = −0.0129989 + 2.97989×	0.996	129.686	11.382	0.128
4	Gallic acid	4.4	168.8	79.0	Neg	*y* = 0.0547697 + 20.8152×	0.999	8.048	16.901	N.D.
6	Protocatechuic acid	6.8	152.8	108.0	Neg	*y* = 0.211373 + 12.8622×	0.957	3.352	2.871	0.145
7	Catechin	7.4	288.8	203.1	Neg	*y* = −0.00370053 + 0.431369×	0.999	N.D.	7.603	N.D.
8	Gentisic acid	8.3	152.8	109.0	Neg	*y* = −0.0238983 + 12.1494×	0.997	N.D.	N.D.	0.016
9	Chlorogenic acid	8.4	353.0	85.0	Neg	*y* = 0.289983 + 36.3926×	0.995	N.D.	N.D.	0.019
10	Protocatechuic aldehyde	8.5	137.2	92.0	Neg	*y* = 0.257085 + 25.4657×	0.996	0.016	0.215	0.357
11	Tannic acid	9.2	182.8	78.0	Neg	*y* = 0.0126307 + 26.9263×	0.999	8.559	0.429	N.D.
15	Epicatechin	11.6	289.0	203.0	Neg	*y* = −0.0172078 + 0.0833424×	0.996	N.D.	45.26	N.D.
16	Vanilic acid	11.8	166.8	108.0	Neg	*y* = −0.0480183 + 0.779564×	0.999	N.D.	0.178	N.D.
17	Caffeic acid	12.1	179.0	134.0	Neg	*y* = 0.120319 + 95.4610×	0.999	N.D.	N.D.	0.17
19	Vanillin	13.9	153.1	125.0	Poz	*y* = 0.00185898 + 20.7382×	0.996	N.D.	N.D.	0.054
20	Syringic aldehyde	14.6	181.0	151.1	Neg	*y* = −0.0128684 + 7.90153×	0.999	N.D.	N.D.	0.024
22	Epicatechin gallate	15.5	441.0	289.0	Neg	*y* = −0.0142216 + 1.06768×	0.997	N.D.	2.395	N.D.
24	*p*-Coumaric acid	17.8	163.0	93.0	Neg	*y* = 0.0249034 + 18.5180×	0.999	0.065	0.074	0.664
29	Salicylic acid	21.8	137.2	65.0	Neg	*y* = 0.239287 + 153.659×	0.999	N.D.	0.024	0.079
30	Cyranoside	23.7	447.0	284.0	Neg	*y* = 0.280246 + 6.13360×	0.997	0.11	0.567	0.181
31	Miquelianin	24.1	477.0	150.9	Neg	*y* = −0.00991585 + 5.50334×	0.999	0.355	0.045	N.D.
33	Rutin	25.6	608.9	301.0	Neg	*y* = −0.0771907 + 2.89868×	0.999	0.085	0.017	0.038
34	isoquercitrin	25.6	463.0	271.0	Neg	*y* = −0.111120 + 4.10546×	0.998	1.444	0.21	0.022
35	Hesperidin	25.8	611.2	449.0	Poz	*y* = 0.139055 + 13.2785×	0.999	0.079	0.017	0.027
37	Genistin	26.3	431.0	239.0	Neg	*y* = 1.65808 + 7.57459×	0.991	0.622	0.074	0.512
39	Ellagic acid	27.6	301.0	284.0	Neg	*y* = 0.00877034 + 0.663741×	0.999	N.D.	0.279	N.D.
40	Cosmosiin	28.2	431.0	269.0	Neg	*y* = −0.708662 + 8.62498×	0.998	0.457	0.059	0.403
41	Quercitrin	29.8	447.0	301.0	Neg	*y* = −0.00153274 + 3.20368×	0.999	6.143	N.D.	0.021
42	Astragalin	30.4	447.0	255.0	Neg	*y* = 0.00825333 + 3.51189×	0.999	1.569	0.53	0.024
43	Nicotiflorin	30.6	592.9	255.0/284.0	Neg	*y* = 0.00499333 + 2.62351×	0.999	0.108	0.048	N.D.
44	Fisetin	30.6	285.0	163.0	Neg	*y* = 0.0365705 + 8.09472×	0.999	0.013	N.D.	N.D.
47	Quercetin	35.7	301.0	272.9	Neg	*y* = +0.00597342 + 3.39417×	0.999	0.269	0.08	1.968
48	Naringenin	35.9	270.9	119.0	Neg	*y* = −0.00393403 + 14.6424×	0.999	0.053	0.05	5.747
49	Hesperetin	36.7	301.0	136.0/286.0	Neg	*y* = +0.0442350 + 6.07160×	0.999	N.D.	N.D.	0.087
50	Luteolin	36.7	284.8	151.0/175.0	Neg	*y* = −0.0541723 + 30.7422×	0.999	0.037	0.01	0.601
52	Kaempferol	37.9	285.0	239.0	Neg	*y* = −0.00459557 + 3.13754×	0.999	0.029	0.019	2.005
53	Apigenin	38.2	268.8	151.0/149.0	Neg	*y* = 0.119018 + 34.8730×	0.998	0.006	0.003	2.471
54	Amentoflavone	39.7	537.0	417.0	Neg	*y* = 0.727280 + 33.3658×	0.992	N.D.	N.D.	0.012
55	Chrysin	40.5	252.8	145.0/119.0	Neg	*y* = −0.0777300 + 18.8873×	0.999	0.04	0.015	17.078
56	Acacetin	40.7	283.0	239.0	Neg	*y* = −0.559818 + 163.062×	0.997	0.058	0.022	62.332

^a^ R.T.: Retention time, ^b^ MI (*m*/*z):* Molecular ions of the standard analytes (*m*/*z* ratio), ^c^ FI (*m*/*z):* Fragment ions ^d^ *r*^2^: Coefficient of determination, N.D.: Not detected.

**Table 3:** Yield percentages, collection localities, and dates of the plant species studied.

Codes	Species Names	Plant Parts	Collection Localities and Dates	Yield (*w*/*w*%)
S1	*Cotinus coggygria*	Commercial leaf extract	Purchased from VemoHerb^®^, Bulgaria, 2020	-
S2	*Cotinus coggygria*	Pedicel	Ankara province, June 2019	13.76
S3	*Cotinus coggygria*	Folia	Ankara province, November 2019	20.77
S4	*Garcinia* × *mangostana*	Pericarpium	Purchased from Thailand, June 2011	21.37
S5	Propolis	Commercial extract	Purchased from Zhejiang Shaoxing Dongling Health Food Co., Ltd., China, 2016	-
S6	*Pistacia vera*	Shell	Gaziantep province, 2019	11.20
S7	*Vitis vinifera*	Seed	Denizli province, 2019	2.45

**Table 4:** The prediction model of the United Nations Globally Harmonized System (UN-GHS) for skin corrosion.

Viability Measured After Exposure Time Points (t = 3 and 60 min)	Prediction to Be Considered
STEP 1	
<50% after 3 min exposure	Corrosive
≥50% after 3 min exposure AND <15% after 60 min exposure	Corrosive
≥50% after 3 min exposure AND ≥15% after 60 min exposure	Non-corrosive
STEP 2	
<15% after 3 min exposure	Optional Sub-category 1A
≥15% after 3 min exposure	A combination of optional Subcategories 1B and 1C

## Data Availability

The original contributions presented in this study are included in the article/Supplementary Materials. Further inquiries can be directed to the corresponding author.

## Supplementary Materials

The following supporting information can be downloaded at https://www.mdpi.com/article/10.3390/ph18030439/s1: Figure S1: NF-κB induction (%) and cell viability values of the *Cotinus coggygria* extracts (Ext 1, Ext 2, and Ext 3), and mangosteen (Ext 4) extract at different concentrations via TNFα addition (mean ± S.D., *n* = 9); Figure S2: NF-κB induction (%) and cell viability values of the propolis (Ext 5), *Pistacia vera* (Ext 6), and grape seed (Ext 7) extract at different concentrations via TNFα addition (mean ± S.D., *n* = 9); Figure S3: NF-κB induction (%) and cell viability values of the *Cotinus coggygria* extracts (Ext 1, Ext 2, and Ext 3), and mangosteen (Ext 4) extract at different concentrations via UV-B irradiation (pre-treatment); Figure S4: NF-κB induction (%) and cell viability values of *Cotinus coggygria* extracts (Ext 1, Ext 2, and Ext 3), and mangosteen (Ext 4) extract at different concentrations via UV-B irradiation (post-treatment); Figure S5: NF-κB induction (%) and cell viability values of the propolis (Ext 5), pistachio (Ext 6), and grape seed (Ext 7) extract at different concentrations via UV-B irradiation (pre-treatment); Figure S6: NF-κB induction (%) and cell viability values of the propolis (Ext 5), pistachio (Ext 6), and grape seed (Ext 7) extract at different concentrations via UV-B irradiation (post-treatment); Figure S7: NF-κB induction (%) and cell viability values of *Cotinus coggygria* extracts (Ext 1, Ext 2, and Ext 3), and mangosteen (Ext 4) extract via H_2_O_2_ stimulation; Figure S8: NF-κB induction (%) and cell viability values of the propolis (Ext 5), pistachio (Ext 6), and grape seed (Ext 7) extract via H_2_O_2_ stimulation; Figure S9: Cytokine/chemokine levels of the subnatants detected with Luminex in two different experiments; Figure S10: Cytokine/chemokine levels of the subnatants detected with Luminex for propolis (Ext 5) and grape seed extract (Ext 7) in two more replicates; Figure S11: The photographs and analysis results using ImageJ software (2 replicates) of epiCS-M after 14 days treatment with S3 (label on images, respectively: 1,2: Control, 3,4: IBMX, 5,6: 4-Butylresorcinol, 1,2: S3 10 µg/mL, 3,4: S3 20 µg/mL, 5,6: S3 30 µg/mL); Figure S12: The photographs and analysis results using ImageJ software (2 replicates) of epiCS-M after 14 days treatment with S5 (labels on images, respectively: 1,2: Control, 3,4: IBMX, 5,6: 4-Butylresorcinol, 1,2: S5 10 µg/mL, 3,4: S5 20 µg/mL, 5,6: S5 30 µg/mL); Figure S13: The photographs and analysis results using ImageJ software (2 replicates) of epiCS-M after 14 days treatment with S7 (labels on images, respectively: 1,2: Control, 3,4: IBMX, 5,6: 4-Butylresorcinol, 1,2: S7 10 µg/mL, 3,4: S7 20 µg/mL, 5,6: S7 30 µg/mL).

## Abbreviations

The following abbreviations are used in this manuscript.

COX-2	Cyclooxygenase-2
FBS	Fetal bovine serum
HaCaT	Human keratinocyte
IBMX	3-Isobutyl-1-methylxanthine
IR	Infrared
iNOS	Nitric oxide synthase
LPS	Lipopolysaccharide
Luc	Luciferase
MMPs	Matrix metalloproteinases
MTT	3-(4,5-dimethylthiazol-2-yl)-5-(3-carboxymethoxyphenyl)-2-(4-sulfophenyl)-2H-tetrazolium
NO	Nitric oxide
NF-κB	Nuclear factor kappa B
RHE	Reconstructed human epidermis
S.D.	Standard deviation
TNFα	Tumor necrosis factor-alpha
TYR	Tyrosinase
UN-GHS	United Nations Globally Harmonized System
WFA	Withaferin A
