# Supplementary material for: Evaluation of the Anti-Aging Properties of Ethanolic Extracts from Selected Plant Species and Propolis by Enzyme Inhibition Assays and 2D/3D Cell Culture Methods
Source: Pharmaceuticals (Basel). 2025 Mar 20;18(3):439. doi: 10.3390/ph18030439 (PMC11944460; doi:10.3390/ph18030439)
Supplement: Supplementary file 1 [file pharmaceuticals-18-00439-s001.zip › pharmaceuticals-3498693-supplementary.pdf]

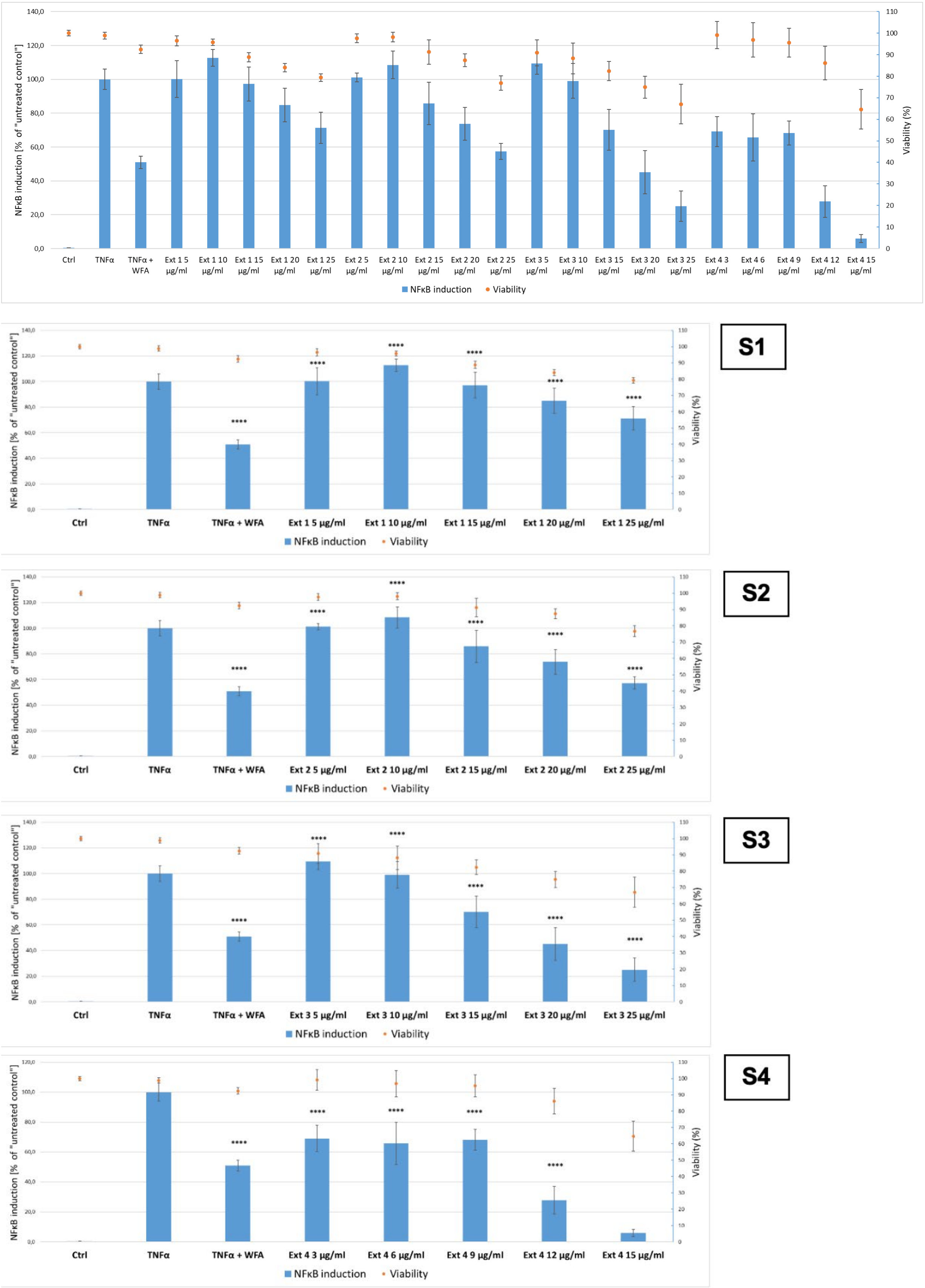

Figure S1. NF-κB induction (%) and cell viability values of the *Cotinus coggygria* extracts (Ext 1, Ext 2, and Ext 3), and mangosteen (Ext 4) extract at different concentrations *via* TNFα addition (Mean ± S.D., n= 9, WFA: withaferin A, ****p<0.0001)

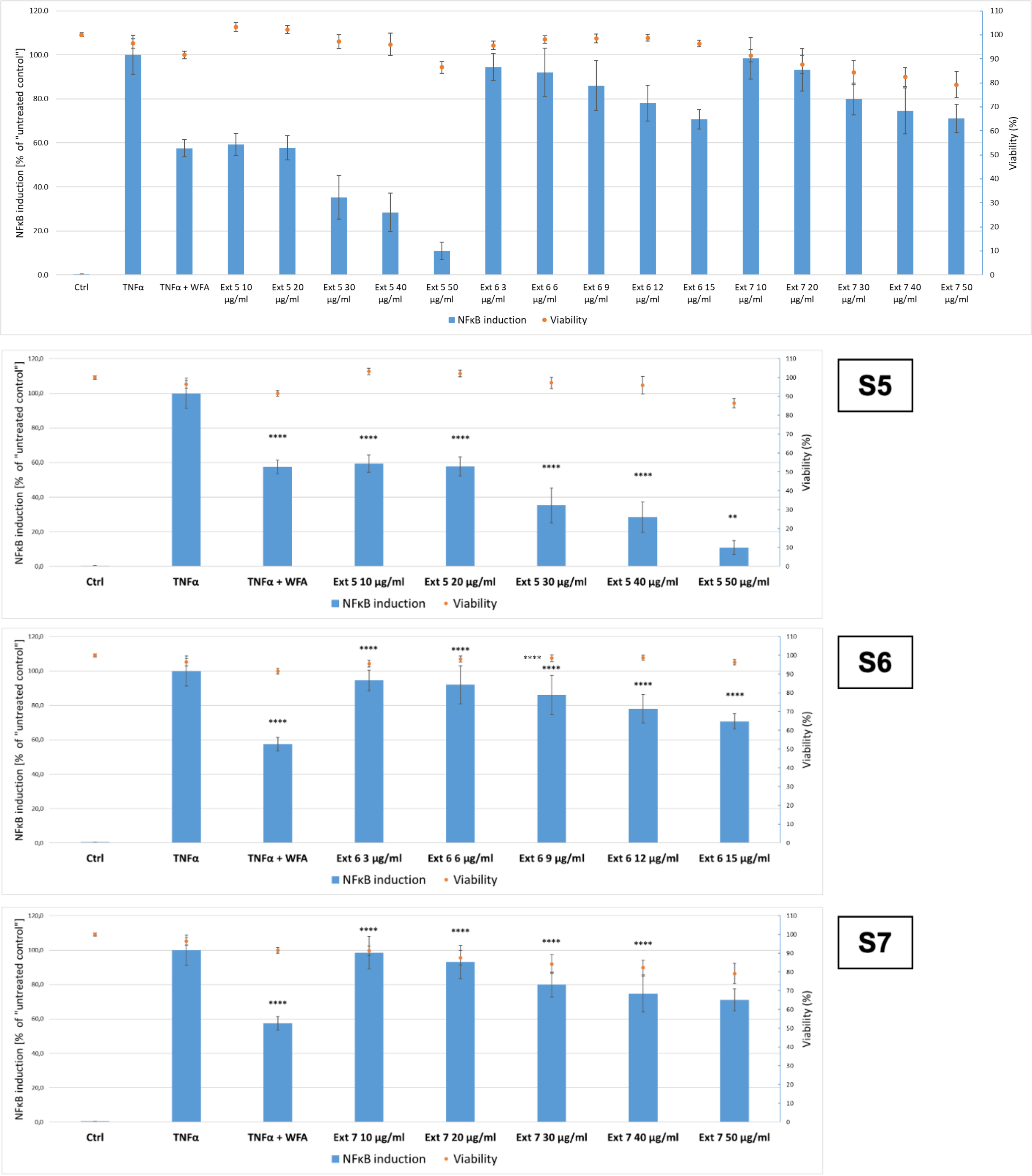

Figure S2. NF-κB induction (%) and cell viability values of the propolis (Ext 5), *Pistacia vera* (Ext 6), and grape seed (Ext 7) extract at different concentrations *via* TNFα addition (Mean ± S.D., n= 9, WFA: withaferin A, **p<0.01, ****p<0.0001)

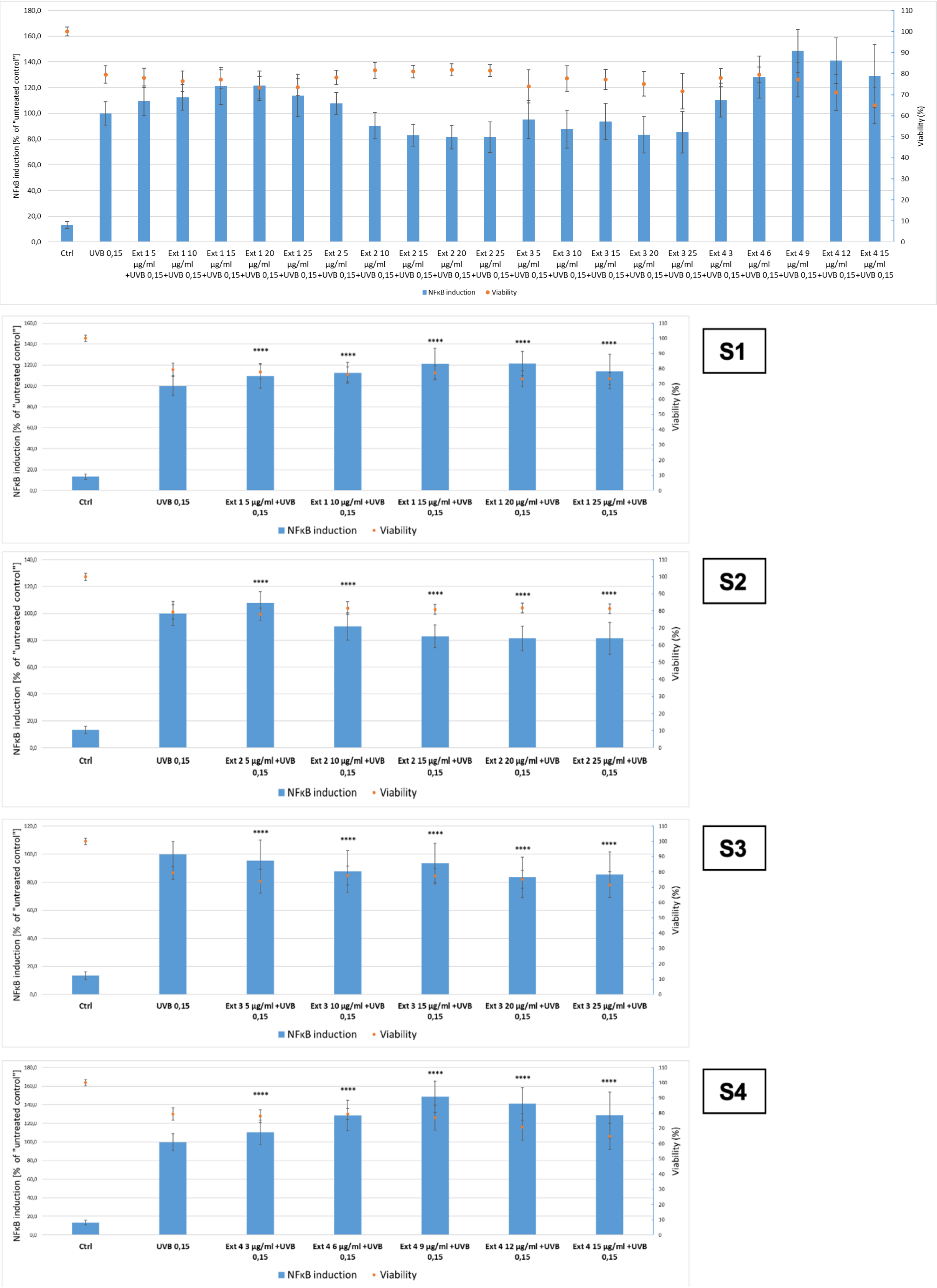

Figure S3: NF-κB induction (%) and cell viability values of the *Cotinus coggygria* extracts (Ext 1, Ext 2, and Ext 3), and mangosteen (Ext 4) extract at different concentrations *via* UV-B irradiation (pre-treatment, ****p<0.0001)

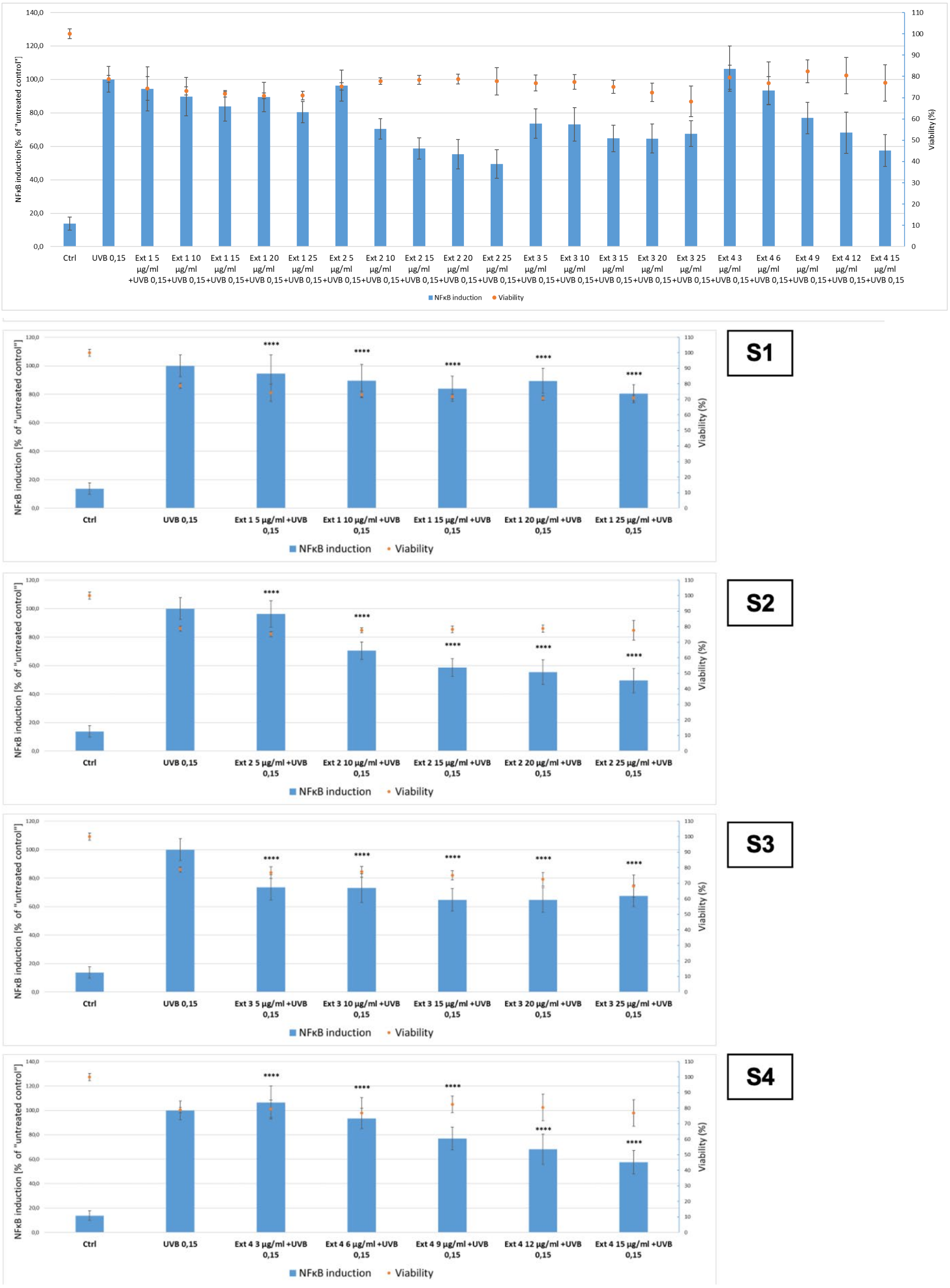

Figure S4: NF-κB induction (%) and cell viability values of *Cotinus coggygria* extracts (Ext 1, Ext 2, and Ext 3), and mangosteen (Ext 4) extract at different concentrations *via* UV-B irradiation (post-treatment, ****p<0.0001)

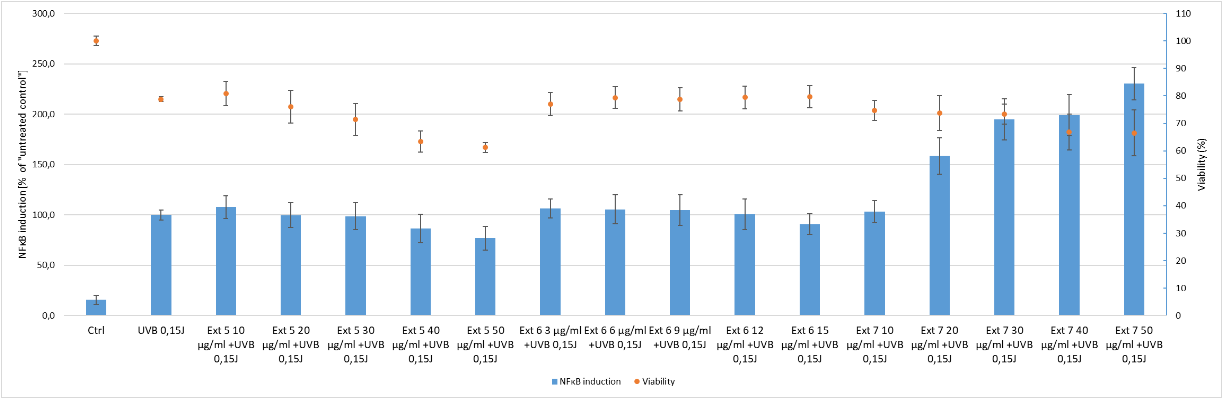

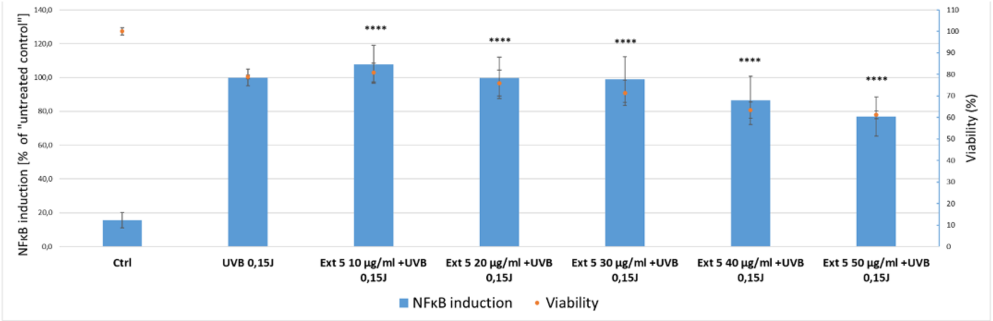

S5

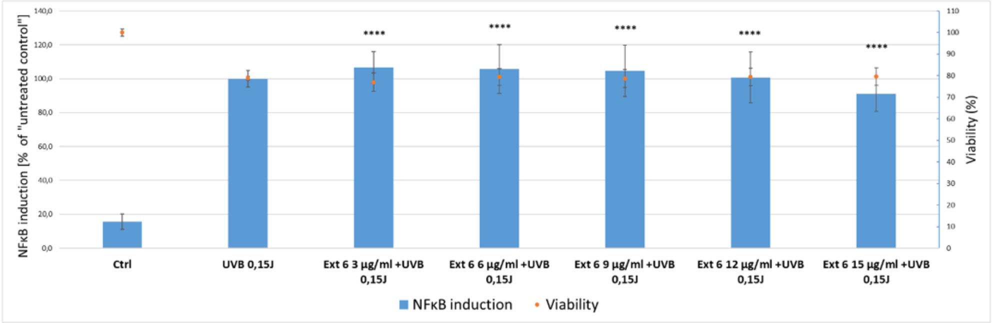

S6

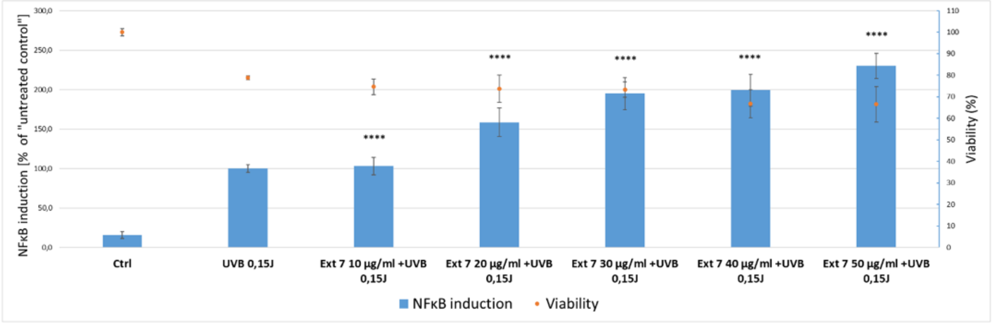

S7

Figure S5: NF-κB induction (%) and cell viability values of the propolis (Ext 5), pistachio (Ext 6), and grape seed (Ext 7) extract at different concentrations *via* UV-B irradiation (pre-treatment, ****p<0.0001)

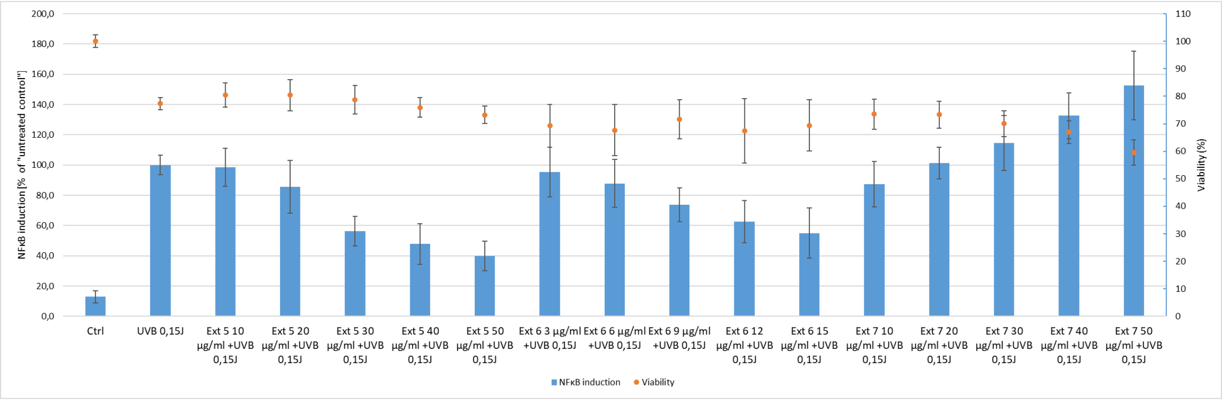

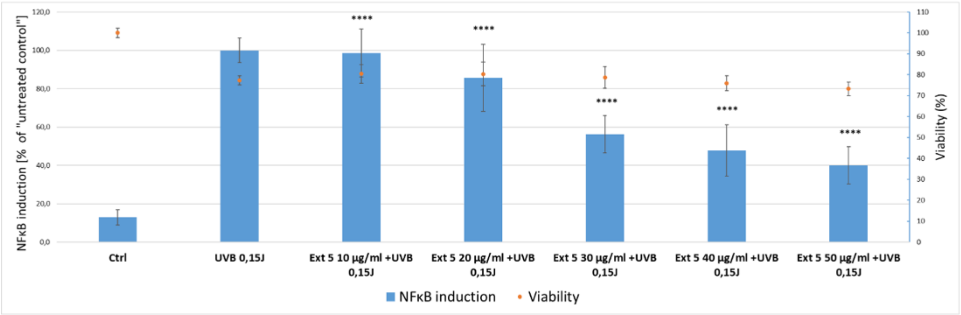

S5

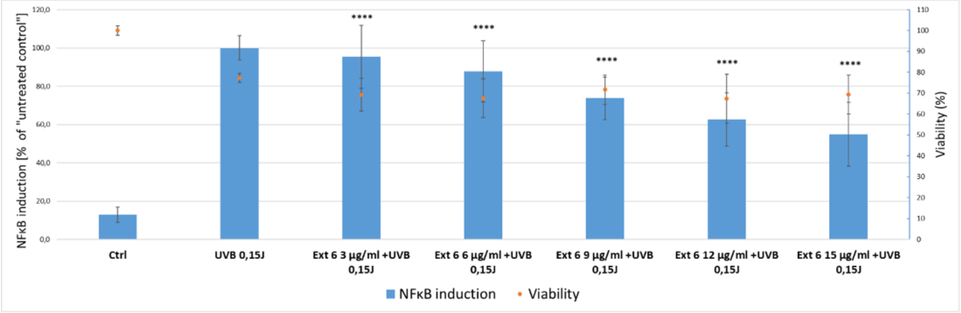

S6

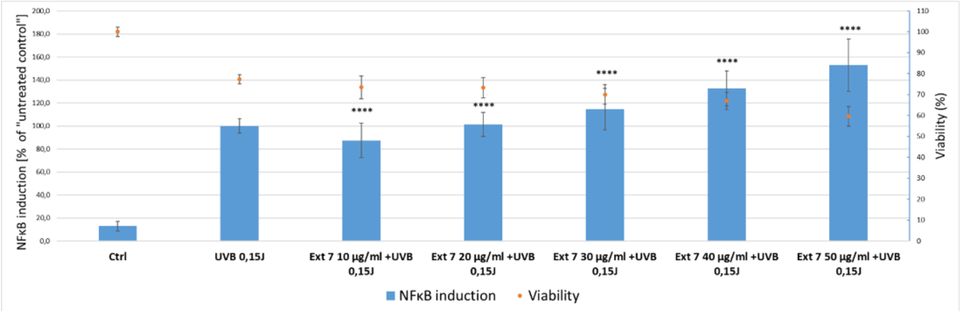

S7

Figure S6: NF-κB induction (%) and cell viability values of the propolis (Ext 5), pistachio (Ext 6), and grape seed (Ext 7) extract at different concentrations *via* UV-B irradiation (post-treatment, ****p<0.0001)

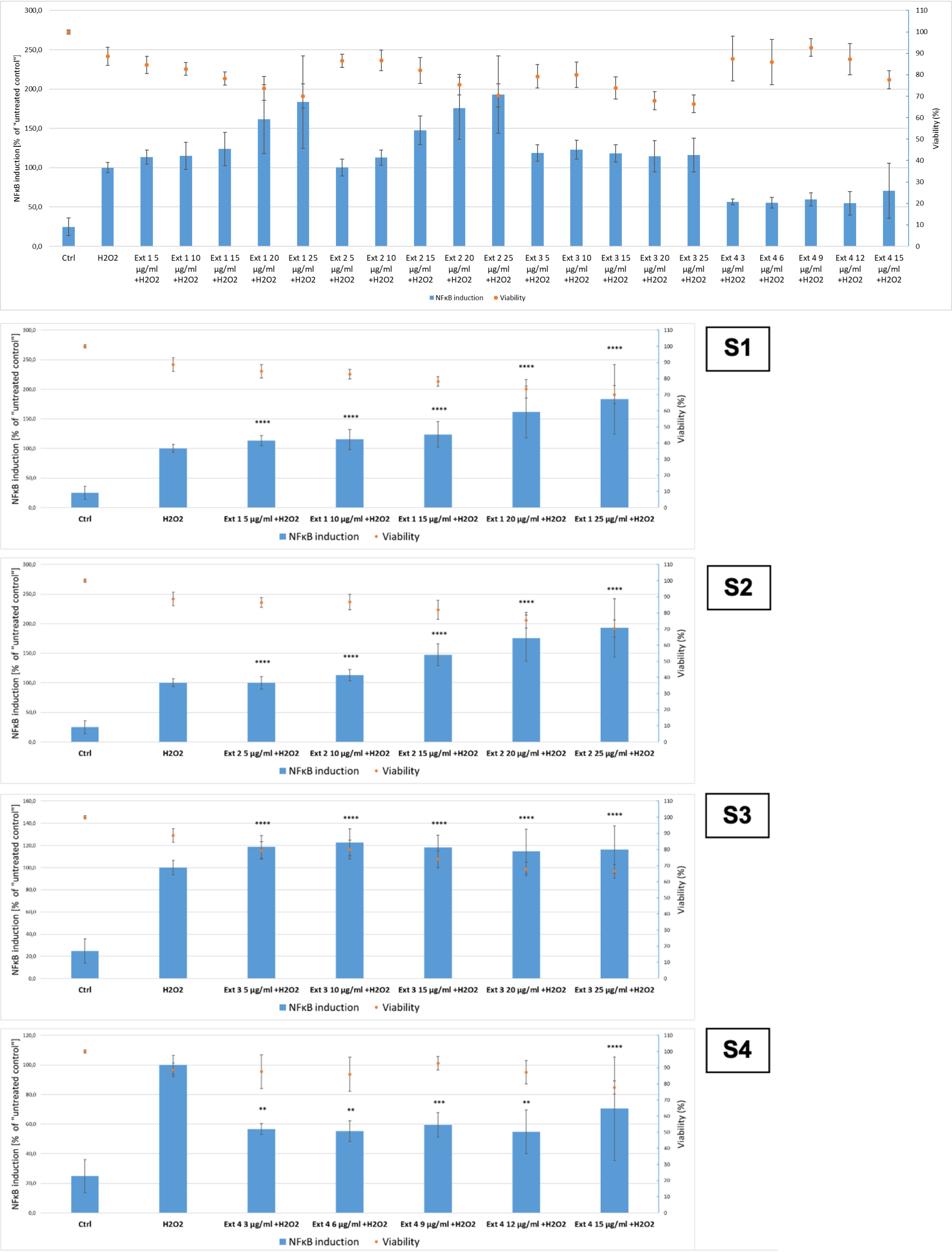

Figure S7: NF-κB induction (%) and cell viability values of *Cotinus coggygria* extracts (Ext 1, Ext 2, and Ext 3), and mangosteen (Ext 4) extract *via* H_2O_2 stimulation (***p*<0.01, ****p*<0.001, *****p*<0.0001)

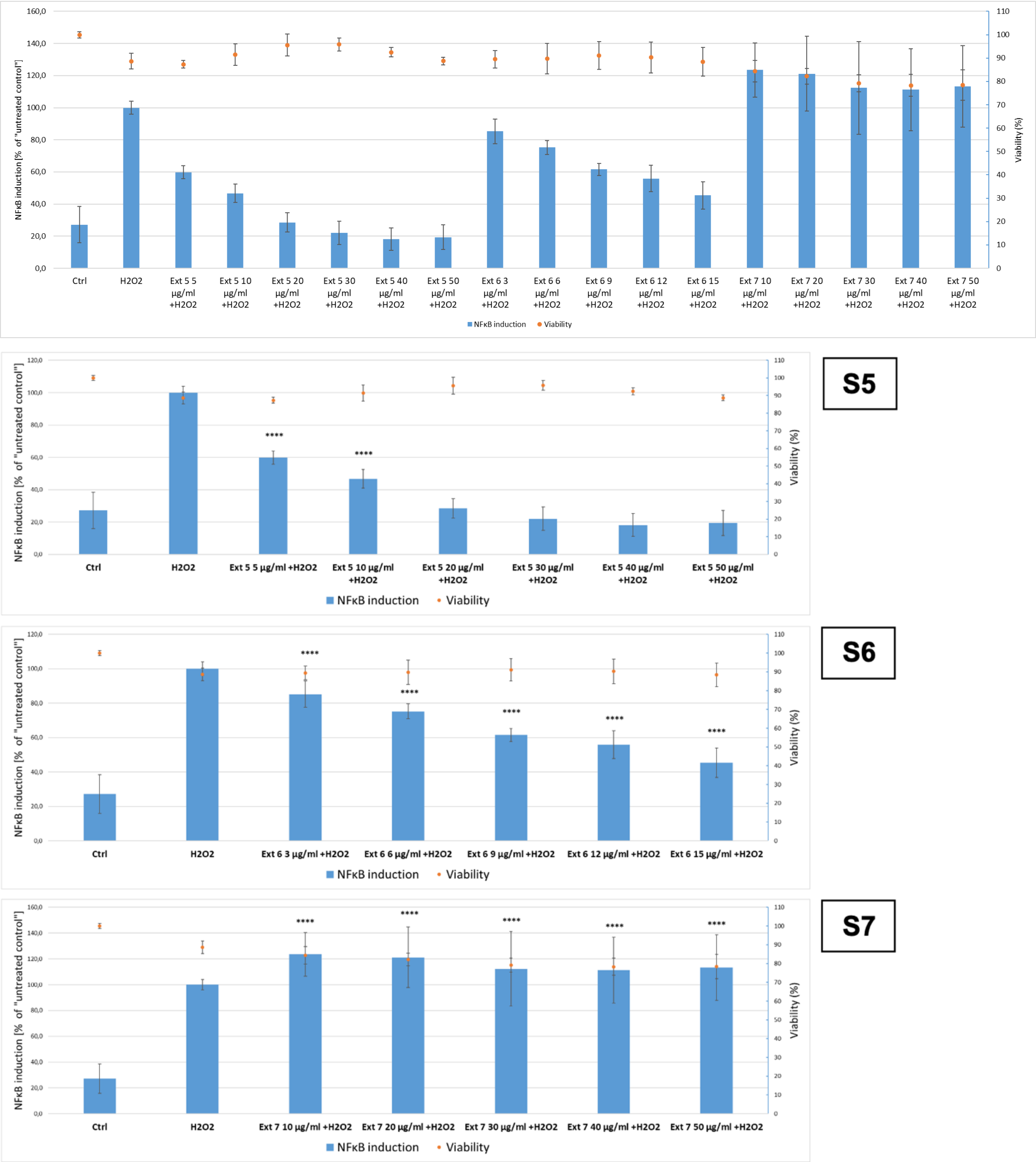

Figure S8: NF-κB induction (%) and cell viability values of the propolis (Ext 5), pistachio (Ext 6), and grape seed (Ext 7) extract *via* H_2O_2 stimulation (*****p*<0.0001)

Sample	TNF-alpha	IP-10	IL-10	MCP-1	RANTES	GM-CSF	TSLP
Standard6	177	83,5	178,25	120,5	610,75	133,5	108
Standard5	604,5	304,75	742,5	1029,5	2475	451	366,5
Standard4	1845,25	1150,25	2427,25	7174	9568	1358,25	1135
Standard3	5491,25	4820	7579,5	35475,5	32255,25	4288,5	3552
Standard2	15616,5	18138,5	21969,75	99827	79023,75	12155,25	10740
Standard1	41612	52936,5	58892,5	142870,5	129010,5	32864,25	29897,75

Sample	TNF-alpha	IP-10	IL-10	MCP-1	RANTES	GM-CSF	TSLP
AIA 76 24h Control	98	12912,5	105	38,5	194,75	156,75	53,5
AIA 76 24h TNFa	90579,25	151384	1469,25	11772	44306	1201,75	615
AIA 76 24h TNFa +WFA 6h	93348,75	153808	1405,75	1954,5	2209,25	1018	239
AIA 76 24h Extr 3 0.125 mg/ml 6h TNFa	91373,5	153612,3	1408,5	25146,25	59845,25	1240,5	972
AIA 76 24h Extr 3 0.25 mg/ml 6h TNFa	91984,25	154677,3	1376	19708,75	65055,5	1289,5	1204
AIA 76 24h Extr 3 0.5 mg/ml 6h TNFa	91281,75	152719,5	1434	24093	53523,75	1212,25	1518
AIA 76 24h Extr 5 0.125 mg/ml 6h TNFa	89181	155241,8	1413,25	20910	52309	1193,5	1548,5
AIA 76 24h Extr 5 0.25 mg/ml 6h TNFa	92917	155098	1450	22435	58198,75	1200,25	1306,5
AIA 76 24h Extr 5 0.5 mg/ml 6h TNFa	91869,25	156306,5	1375	14456,25	41617,25	1112,25	1654,5
AIA 76 24h Extr 7 0.125 mg/ml 6h TNFa	91696,25	156749,8	1365	22448,25	43943,25	1187,75	1279,25
AIA 76 24h Extr 7 0.25 mg/ml 6h TNFa	93970,75	154003,5	1470,5	33080,25	53023	1167,5	1381,25
AIA 76 24h Extr 7 0.5 mg/ml 6h TNFa	91794,5	154481,5	1328,25	30265,25	44547,5	1255,25	1736

Sample	TNF-alpha	IP-10	IL-10	MCP-1	RANTES	GM-CSF	TSLP
AIA 76 48h Control	56	1190,5	6	231	111	137	10,5
AIA 76 48h TNFa	3857,75	70347,25	469,25	549	13857	594,5	73
AIA 76 48h TNFa +WFA 6h	3885,25	42285,5	326,75	198	573	360,5	29
AIA 76 48h Extr 3 0.125 mg/ml 6h TNFa	3273,5	104166,5	712,75	1104,25	19813,5	594,5	70
AIA 76 48h Extr 3 0.25 mg/ml 6h TNFa	3422,5	97442,25	693,75	912,75	31086,75	574	96,25
AIA 76 48h Extr 3 0.5 mg/ml 6h TNFa	3522,25	101553	678,75	1394,5	18374	585,25	99,25
AIA 76 48h Extr 5 0.125 mg/ml 6h TNFa	4123	103718	711,25	1075,25	14584	655,25	103,75
AIA 76 48h Extr 5 0.25 mg/ml 6h TNFa	3943,5	101230,5	735,75	554,5	17064,5	638,25	108,75
AIA 76 48h Extr 5 0.5 mg/ml 6h TNFa	3891,75	87954	676,75	384,25	11763	505,5	140
AIA 76 48h Extr 7 0.125 mg/ml 6h TNFa	3734,75	112100,5	759,5	1153,25	12548	683,5	74
AIA 76 48h Extr 7 0.25 mg/ml 6h TNFa	5533	126748	865,5	3275,25	18777	694	110,5
AIA 76 48h Extr 7 0.5 mg/ml 6h TNFa	4179,75	110949,3	746	1626,5	13491,5	632	96

Sample	TNF-alpha	IP-10	IL-10	MCP-1	RANTES	GM-CSF	TSLP
Standard6	181	103	190	111	509	100	88
Standard5	551	386	641	900	2094	332	271
Standard4	1733	1739	2262	6583	8666	1089	864
Standard3	5052	7032	7563	32842	28344	3469	2697
Standard2	14427	26095	23126	97821	72529	10701	8476
Standard1	40191	70552	64022	140266	120055	28749	24987

Sample	TNF-alpha	IP-10	IL-10	MCP-1	RANTES	GM-CSF	TSLP
AIA 77 24h Control	31	512	2	36	57	44	14
AIA 77 24h TNFa	91391	137172	1169	6386	6171	819	507
AIA 77 24h TNFa +WFA 6h	91863	98176	767	785	239	476	132
AIA 77 24h Extr 3 0.125 mgml 6h TNFa	90573	145587	1326	11734	8778	872	624
AIA 77 24h Extr 3 0.25 mgml 6h TNFa	90136	133203	1086	5566	6309	802	374
AIA 77 24h Extr 3 0.5 mgml 6h TNFa	91310	141659	1257	12439	8734	931	420
AIA 77 24h Extr 5 0.125 mgml 6h TNFa	89588	144014	1356	10860	5862	828	438
AIA 77 24h Extr 5 0.25 mgml 6h TNFa	89462	141607	1282	5384	5248	805	393
AIA 77 24h Extr 5 0.5 mgml 6h TNFa	90239	138853	1227	3473	3498	790	468
AIA 77 24h Extr 7 0.125 mgml 6h TNFa	92854	144878	1295	15687	6884	837	390
AIA 77 24h Extr 7 0.25 mgml 6h TNFa	94277	140754	1321	10737	9407	923	441
AIA 77 24h Extr 7 0.5 mgml 6h TNFa	92105	132972	1122	4853	4017	839	306

Sample	TNF-alpha	IP-10	IL-10	MCP-1	RANTES	GM-CSF	TSLP
AIA 77 48h Control	45	987	3	477	34	60	5
AIA 77 48h TNFa	4261	31026	310	591	2197	266	31
AIA 77 48h TNFa +WFA 6h	3214	12799	115	97	127	111	4
AIA 77 48h Extr 3 0.125 mgml 6h TNFa	3789	48024	419	777	2314	319	30
AIA 77 48h Extr 3 0.25 mgml 6h TNFa	3708	21871	199	356	1895	188	29
AIA 77 48h Extr 3 0.5 mgml 6h TNFa	3040	36873	323	714	2168	249	23
AIA 77 48h Extr 5 0.125 mgml 6h TNFa	3152	35623	320	518	1598	296	31
AIA 77 48h Extr 5 0.25 mgml 6h TNFa	3509	28330	255	220	1369	225	29
AIA 77 48h Extr 5 0.5 mgml 6h TNFa	3387	27346	249	141	729	208	34
AIA 77 48h Extr 7 0.125 mgml 6h TNFa	3532	59849	508	1329	2535	320	23
AIA 77 48h Extr 7 0.25 mgml 6h TNFa	4478	48007	407	975	3001	301	23
AIA 77 48h Extr 7 0.5 mgml 6h TNFa	3863	23752	227	451	1492	291	27

Figure S9: Cytokine/chemokine levels of the subnatants detected with Luminex in two different experiments

Sample	TNF-alpha	IP-10	IL-10	MCP-1	RANTES	GM-CSF	TSLP
Standard6	186	121,5	184,75	94	600	118,5	89,25
Standard5	594	423	647	776,5	2219,75	373,25	305,75
Standard4	1840	1904,5	2328,5	6049,5	9145,5	1218,75	1011,5
Standard3	5263	7392	7023,75	31783,25	29048	3627	3001,5
Standard2	15019	25311	21404,5	93164,5	72401,75	10751,5	8915
Standard1	41943	66333	57780,25	137543,3	118709,5	28340,25	24983,25

Sample	TNF-alpha	IP-10	IL-10	MCP-1	RANTES	GM-CSF	TSLP
AIA 80 24h Control	94	17267,75	181	161	371,25	192	66
AIA 80 24h TNFa	93944	148838	1700	61855	48859,75	1311,5	1958,25
AIA 80 24h TNFa + WFA 6h	95056	144877	1410,5	5066,5	1433	955	389,5
AIA 80 24h Ext 5 0.125mg/ml 6h TNFa	95332	149736,5	1656,5	24707,5	27585,5	1271,5	2346,25
AIA 80 24h Ext 5 0.25mg/ml 6h TNFa	93507	150214	1604,75	22904,75	38029,5	1248	2462,25
AIA 80 24h Ext 5 0.5mg/ml 6h TNFa	91987	149788,3	1621,25	9047	22369,5	1090,5	2336,25
AIA 80 24h Ext 7 0.5mg/ml 6h TNFa	93565	148700,3	1679,25	26377,5	39394	1212,75	1990

Sample	TNF-alpha	IP-10	IL-10	MCP-1	RANTES	GM-CSF	TSLP
AIA 80 48h Control	54	4748,5	39,5	1557	153,5	208,5	12
AIA 80 48h TNFa	2994	125595	1081,5	4939,75	19006,5	745	110,5
AIA 80 48 TNFa + WFA 6h	3480	59551	505,75	908	573	370,75	40,75
AIA 80 48h Ext 5 0.125mg/ml 6h TNFa	3616	96865	851,75	2100,75	11975	551,75	186,25
AIA 80 48h Ext 5 0.25mg/ml 6h TNFa	3374	91988,75	748,75	1213,25	16285,5	513	228,75
AIA 80 48h Ext 5 0.5mg/ml 6h TNFa	3471	61249,5	531,75	635,25	8912,25	511	206,25
AIA 80 48h Ext 7 0.5mg/ml 6h TNFa	3271	105501,5	878,25	2178,75	18158	549,25	155,25

Sample	TNF-alpha	IP-10	IL-10	MCP-1	RANTES	GM-CSF	TSLP
AIA 80 24h Control	23	1455,75	0	4,25	138,25	58,5	11,75
AIA 80 24h TNFa	92033	148544,8	830,5	4037,5	45077	1066,5	864,25
AIA 80 24h TNFa + WFA 6h	94255	131738,5	1154,75	177,75	973,75	692,5	248,25
AIA 80 24h Ext 7 0.125mg/ml 6h TNFa	93340	147606,3	1656	1983,75	32415,75	1127	956,25
AIA 80 24h Ext 7 0.25mg/ml 6h TNFa	90732	149333,3	1616,25	2520,25	44040,5	1219,5	1253
AIA 80 24h Ext 7 0.5mg/ml 6h TNFa	89258	145027	1519,5	2619	35870,75	1141,5	1058,75
AIA 80 24h Ext 5 0.5mg/ml 6h TNFa	89459	145608,3	1494	380,5	23167,25	1072,75	1565,25

Sample	TNF-alpha	IP-10	IL-10	MCP-1	RANTES	GM-CSF	TSLP
AIA 81 48h Control	12	1075,5	1	14,25	123	41,5	1,25
AIA 81 48h TNFa	3321	123344	997	133,5	12770,75	586	45,25
AIA 81 48 TNFa + WFA 6h	2940	26577,5	219	8,25	312,25	177,25	8,25
AIA 81 468h Ext 7 0.125mg/ml 6h TNFa	4681	93502,75	750,5	64,5	11112,75	523,25	86,75
AIA 81 48h Ext 7 0.25mg/ml 6h TNFa	4120	103883,8	821,25	69	12926,25	620,25	58,5
AIA 81 48h Ext 7 0.5mg/ml 6h TNFa	4297	112548,8	876,25	77,5	10683,5	572,5	91,75
AIA 81 48h Ext 5 0.5mg/ml 6h TNFa	4936	52711,25	417,25	17	6836,5	427	152

Figure S10: Cytokine/chemokine levels of the subnatants detected with Luminex for propolis (Ext 5) and grape seed extract (Ext 7) in two more replicates

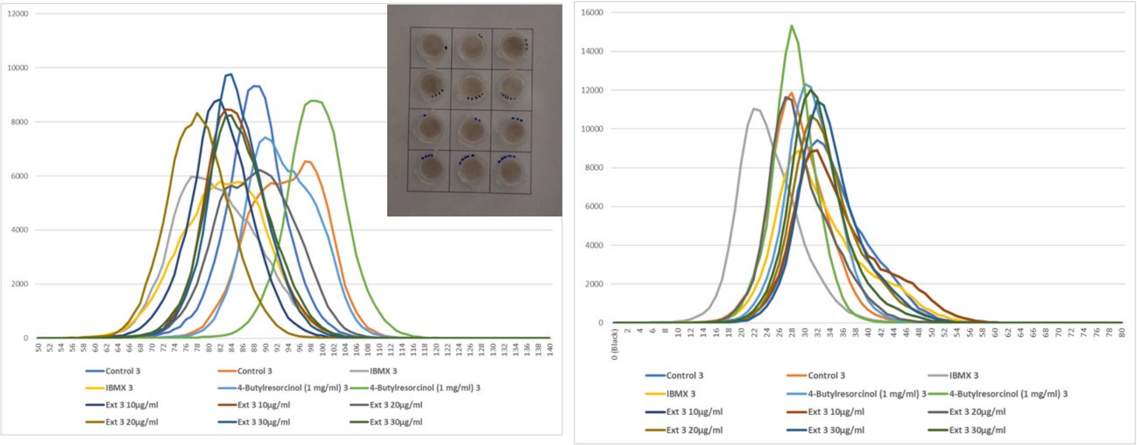

Figure S11: The photographs and analyze results using ImageJ software (2 replicates) of epiCS-M after 14 days treatment with S3 (Label on images respectively; 1,2: Control, 3,4: IBMX, 5,6: 4-Butylresorcinol, 1,2: S3 10 µg/mL, 3,4: S3 20 µg/mL, 5,6: S3 30 µg/mL)

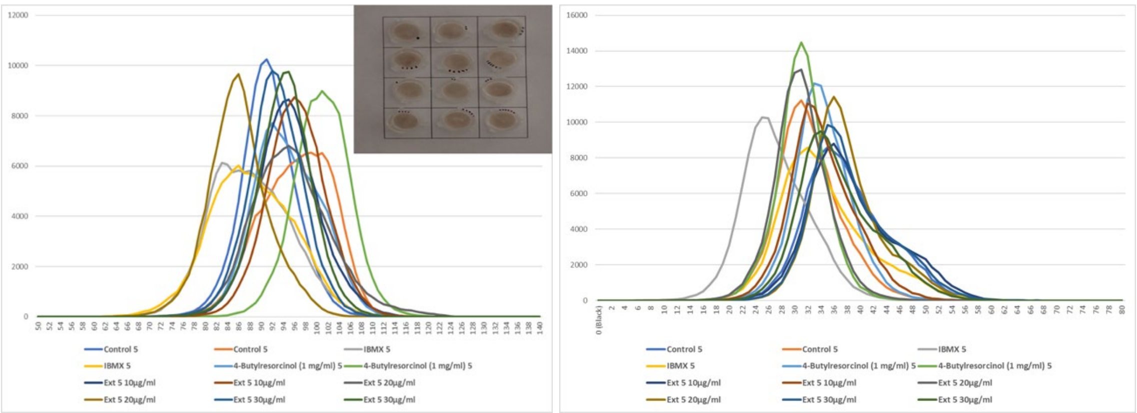

Figure S12: The photographs and analyze results using ImageJ software (2 replicates) of epiCS-M after 14 days treatment with S5 (Labels on images respectively; 1,2: Control, 3,4: IBMX, 5,6: 4-Butylresorcinol, 1,2: S5 10 µg/mL, 3,4: S5 20 µg/mL, 5,6: S5 30 µg/mL)

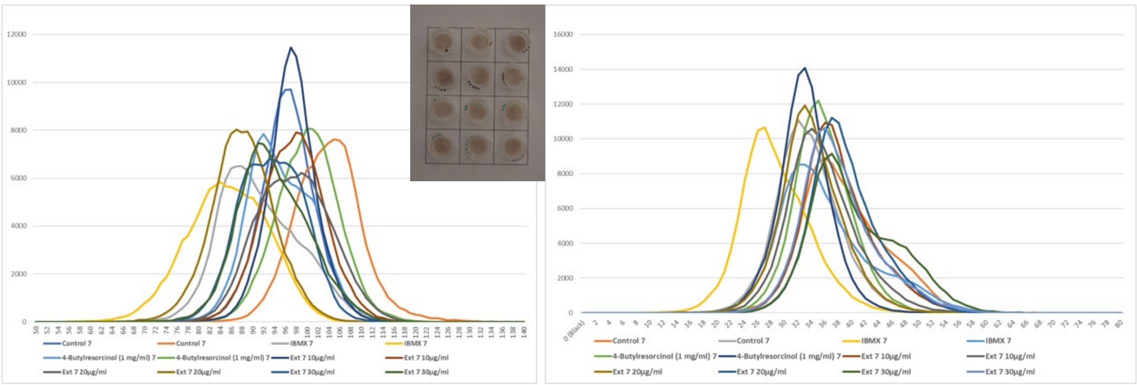

Figure S13: The photographs and analyze results using ImageJ software (2 replicates) of epiCS-M after 14 days treatment with S7 (Labels on images respectively; 1,2: Control, 3,4: IBMX, 5,6: 4-Butylresorcinol, 1,2: S7 10 µg/mL, 3,4: S7 20 µg/mL, 5,6: S7 30 µg/mL)
